# A fibrin biofilm covers blood clots and protects from microbial invasion

**DOI:** 10.1172/JCI98734

**Published:** 2018-06-25

**Authors:** Fraser L. Macrae, Cédric Duval, Praveen Papareddy, Stephen R. Baker, Nadira Yuldasheva, Katherine J. Kearney, Helen R. McPherson, Nathan Asquith, Joke Konings, Alessandro Casini, Jay L. Degen, Simon D. Connell, Helen Philippou, Alisa S. Wolberg, Heiko Herwald, Robert A.S. Ariëns

**Affiliations:** 1 Thrombosis and Tissue Repair Group, Discovery and Translational Science Department, Leeds Institute of Cardiovascular and Metabolic Medicine, School of Medicine, University of Leeds, Leeds, United Kingdom.; 2 Division of Infection Medicine, Department of Clinical Sciences, Faculty of Medicine, Lund University, Lund, Sweden.; 3 Population and Clinical Sciences Department, Leeds Institute of Cardiovascular and Metabolic Medicine, School of Medicine, University of Leeds, Leeds, United Kingdom.; 4 Department of Biochemistry, Cardiovascular Research Institute Maastricht, School of Medicine, and; 5 Synapse Research Institute, CARIM, University of Maastricht, Maastricht, Netherlands.; 6 Division of Angiology and Haemostasis, Faculty of Medicine, Geneva University Hospitals, Geneva, Switzerland.; 7 Cincinnati Children’s Hospital, Cincinnati, Ohio, USA.; 8 Molecular and Nanoscale Physics Group, School of Physics and Astronomy, University of Leeds, Leeds, United Kingdom.; 9 Department of Pathology and Laboratory Medicine, University of North Carolina, Chapel Hill, North Carolina, USA.

**Keywords:** Hematology, Vascular Biology, Coagulation

## Abstract

Hemostasis requires conversion of fibrinogen to fibrin fibers that generate a characteristic network, interact with blood cells, and initiate tissue repair. The fibrin network is porous and highly permeable, but the spatial arrangement of the external clot face is unknown. Here we show that fibrin transitioned to the blood-air interface through Langmuir film formation, producing a protective film confining clots in human and mouse models. We demonstrated that only fibrin is required for formation of the film, and that it occurred in vitro and in vivo. The fibrin film connected to the underlying clot network through tethering fibers. It was digested by plasmin, and formation of the film was prevented with surfactants. Functionally, the film retained blood cells and protected against penetration by bacterial pathogens in a murine model of dermal infection. Our data show a remarkable aspect of blood clotting in which fibrin forms a protective film covering the external surface of the clot, defending the organism against microbial invasion.

## Introduction

Hemostasis is a pivotal mechanism to prevent life-threatening blood loss from sites of injury, and involves close interplay between coagulation and platelets. The resulting blood clot contains activated platelets, red blood cells, and fibrin polymer, which holds the clot together. Fibrin is formed by limited proteolysis of fibrinogen by thrombin. Thrombin cleaves small peptidic sequences from the N-termini of the Aα- and Bβ-polypeptides, exposing interaction sites for binding pockets in the C-terminal domains of the β- and γ-chains, respectively (1). Fibrin thus polymerizes into protofibrils, which aggregate laterally into fibers. The fibers branch and produce a 3D network with remarkable elastic properties (2). The structure of the fibrin network is determined by fiber diameter, protofibril packing, and pore size, the latter of which is sufficient for the incorporation of cells. Dense networks with thin fibers and increased rigidity are associated with increased risk of thrombosis, while loose clot networks with thick fibers and reduced rigidity are associated with bleeding (1, 3, 4).

A major conundrum after decades of fibrin polymer research is that fibrin fibers in blood clots appear endless, with little evidence of fiber ends (Supplemental Figure 1; supplemental material available online with this article; https://doi.org/10.1172/JCI98734DS1). Thus, the mechanisms and structures that determine the external boundary of an extravascular (hemostatic) blood clot are unknown. What happens to the fibrin network and fibers when they reach the peripheral, exposed surface of the clot? Do the fibers just stop, flatten, or bend, or is the surface lined by platelets, red blood cells, or other cells? The architecture of this interface is important because it forms a distinction between host and the environment. To explore this, we investigated the structural characteristics of the exterior face of the blood clot and found that fibrin at the blood-air interface is not arranged in the form of fibers, but instead transitions to a continuous sheet that covers the clot surface. This sheet arises through Langmuir film formation, provides a natural limit to clot growth, prevents blood cell loss, and protects from bacterial infection. These data reveal a critical role for fibrin in hemostasis through the generation of a previously unrecognized bioprotective film that encapsulates the clot in the early stages of tissue repair.

## Results

### Film forms at the air-liquid interface.

**Figure 1:**
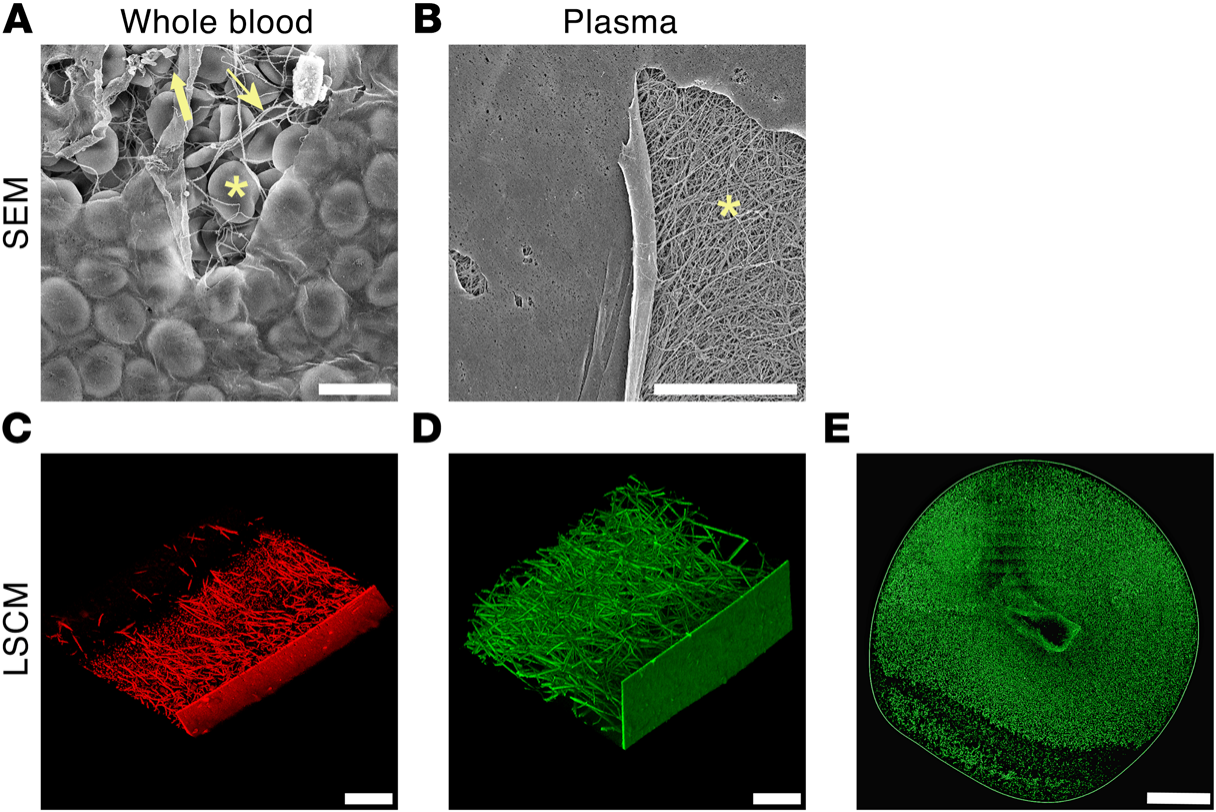
Film forms on the clot surface at the air-liquid interface. (**A** and **B**) SEM of film formed at the air-liquid interface in whole blood and plasma clots. At places where the film is torn (due to SEM sample processing procedures), red blood cells (asterisk), platelets (thick arrow), and fibrin (thin arrow) are observed in whole blood clots (**A**), and fibrin (asterisk) in plasma clots (**B**). Red blood cells are also visible through the film in whole blood clots. (**C** and **D**) LSCM of film formed at the air-liquid interface in whole blood and plasma clots. Fibrinogen was fluorescently labeled with Alexa Fluor 488 (green) or Alexa Fluor 594 (red). Under fully hydrated conditions of LSCM, tears in the film were not observed. (**E**) LSCM of a single *Z*-plane slice of a plasma clot showing continuous film around the clot. The central gap in the clot image is where the pipette was introduced into the plasma drop to inject thrombin. Images represent findings reproduced in at least *n* = 3 experiments. Scale bars: **A** and **B**, 10 μm; **C** and **D**, 50 μm; **E**, 1 mm. All images are representative of *n* = 3 experiments.

Our study was motivated by observations from scanning electron microscopy (SEM) revealing a thin film on the surface of clots where, prior to dehydration, the air-liquid interface had been. This film was present in clots formed from human whole blood or platelet-poor plasma (PPP) when clotting was initiated with thrombin (0.5 U/ml; Figure 1, A and B) or tissue factor (5 pM; Supplemental Figure 2, A and B). Using laser scanning confocal microscopy (LSCM), we found the film was also present in clots from whole blood (thrombin, Figure 1C; tissue factor, Supplemental Figure 2C) and plasma (thrombin, Figure 1D; tissue factor, Supplemental Figure 2D) in fully hydrated conditions. When single slices of a clot in the *z*-plane were imaged using LSCM, the film appeared continuous around the clot exterior (Figure 1E).

### Film is composed of fibrin.

**Figure 2:**
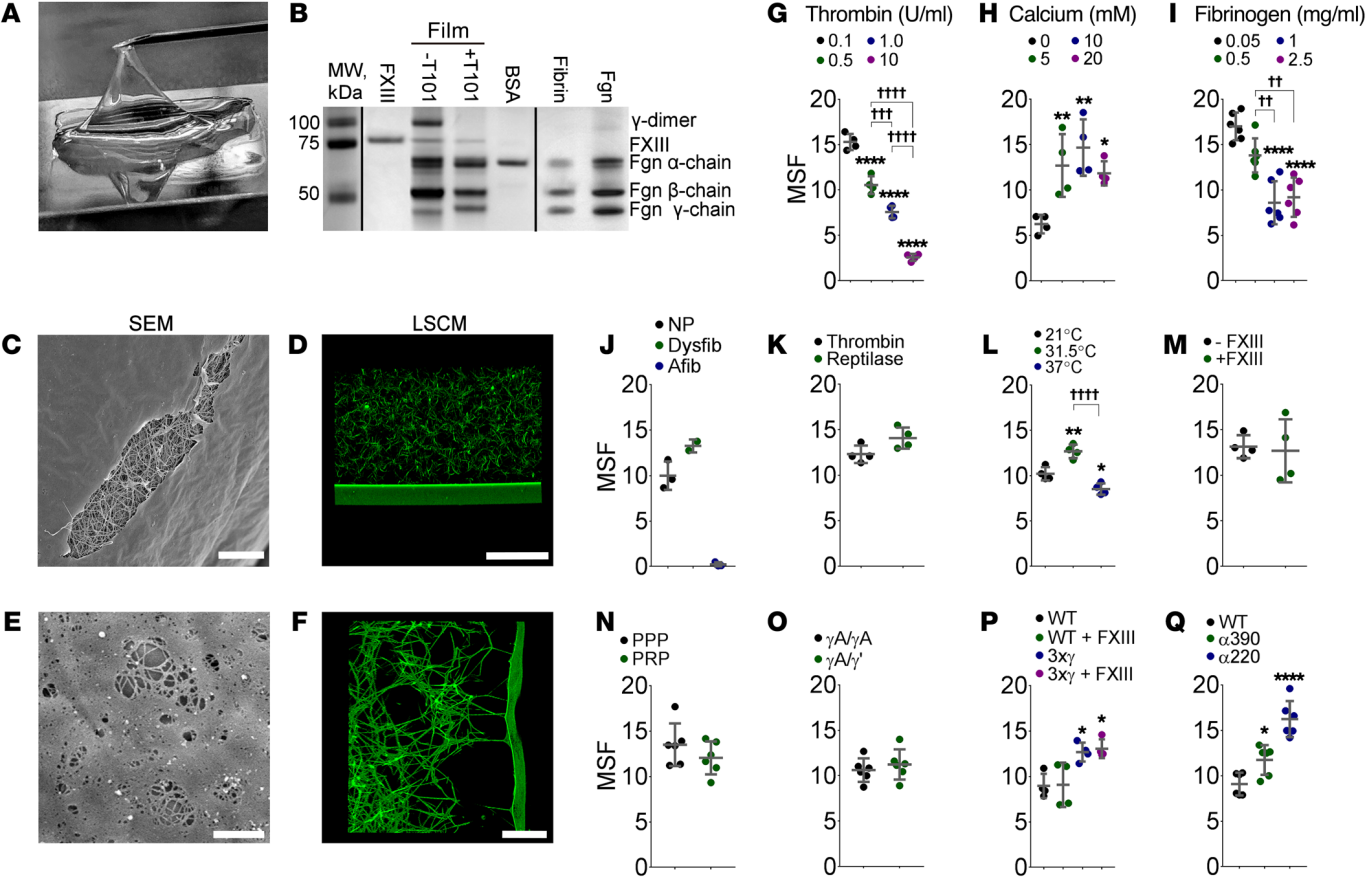
Film only requires fibrin for formation. (**A**) The film was peeled from clots using a needle. (**B)** Films removed from clots produced with or without T101 were run alongside FXIII, BSA, purified fibrin, and purified fibrinogen (Fgn) on reducing SDS-PAGE. MW, molecular weight marker. (**C**) SEM of the film in a clot produced from purified fibrinogen. A tear in the film exposes the underlying clot, as was often observed in SEM. Scale bar: 10 μm. (**D**) LSCM of the film at the air-liquid interface of a clot produced from purified fibrinogen, without breaks in fully hydrated conditions. Fibrinogen fluorescently labeled with Alexa Fluor 488. Scale bar: 50 μm. (**E**) SEM image of areas of film transitioning into fibers. Scale bar: 1 μm. (**F**) LSCM of tethering fibers connected to the film. Scale bar: 50 μm. Images in **A**–**F** are representative of *n* = 3 experiments. (**G**–**Q**) Mean sheet fluorescence (MSF) measurements of the film comparing different conditions: (**G**) thrombin concentration, *n* = 4 experiments; (**H**) CaCl_2_ concentration, *n* = 4 experiments; (**I**) fibrinogen concentration, *n* = 6 experiments; (**J**) normal pool (NP; *n* = 3 patients) versus dysfibrinogenemia (Dysfib, *n* = 2 patients) and afibrinogenemia (Afib, *n* = 3 patients); (**K**) thrombin versus reptilase, *n* = 4 experiments; (**L**) temperature, *n* = 4 experiments; (**M**) FXIII, *n* = 4 experiments; (**N**) PPP versus PRP, *n* = 6 individuals; (**O**) γA/γA fibrinogen versus γA/γ′ fibrinogen, *n* = 6 experiments; (**P**) fibrinogen triple γ-chain crosslinking mutant (3xγ), *n* = 6 experiments; (**Q**) fibrinogen α-chain deletion mutants (α390 and α220), *n* = 6 experiments. Asterisks indicate difference from first column; crosses, difference between other columns. *P < 0.05; **P < 0.01, ^††^*P* < 0.01; ^†††^*P* < 0.001; ****P < 0.0001, ^††††^*P* < 0.0001. Mean ± SD. Unpaired *t* test (2 groups); ANOVA (multiple groups).See Supplemental Table 1 for estimated corresponding film thicknesses.

Fluorescence detected in the LSCM images indicated that fibrinogen or fibrin was present in the film (Figure 1, C–E). To better characterize the film components, we peeled it away from the surface of plasma clots (Figure 2A) formed with or without T101 (transglutaminase inhibitor). The films were reduced and subjected to SDS-PAGE (Figure 2B). The film contained fibrin, and this was confirmed by Western blotting with a polyclonal antibody against fibrinogen (Supplemental Figure 3A).

To assess whether the film could form from fibrin alone, we formed clots from purified fibrinogen (1 mg/ml) and thrombin (0.5 U/ml) and examined them using SEM and LSCM. We observed formation of films that mimicked those formed on whole blood and plasma clots (Figure 2, C and D). Films transitioned to fibers in places (Figure 2E) and connected to the fibrin network through tethering fibers (Figure 2F). These observations show that fibrin(ogen) alone is capable of producing a film at the clot surface.

We next investigated how other plasma components influenced film formation. Purified fibrinogen was spiked with Alexa Fluor 488–fibrinogen (25 μg/ml), and different clotting conditions were tested. Film mean sheet fluorescence (MSF) and film thickness were measured to quantify changes (Supplemental Table 1).

The film formed at all thrombin concentrations (0.1, 0.5, 1, 10 U/ml; Figure 2G and Supplemental Figure 3B). However, as the thrombin concentration increased, the MSF and thickness of the film decreased in a stepwise manner, indicating a reduction in the quantity of fibrin present. Thrombin generation triggered by tissue factor in plasma starts slowly after a short lag phase, and then its concentration rapidly increases to a maximum of several hundred nanomoles (tens of units per milliliter) (5, 6). However, fibrin formation occurs at just 2–3 nM thrombin (0.2–0.3 U/ml), before the vast majority of thrombin has been generated (7). Our data indicate that the film forms most optimally at lower thrombin concentrations, which led to longer clotting times, allowing more fibrin to accumulate at the surface before the underlying network has formed. Previous data showed that lower thrombin concentrations are also optimal for maximum protofibril packing in fibrin fibers (8).

CaCl_2_ concentration (5, 10, 20 mM) had no effect on film formation (Figure 2H and Supplemental Figure 3C), but the absence of CaCl_2_ resulted in at least a 1.9-fold decrease in MSF and 1.5-fold decrease in film thickness. This indicates that film formation is not dependent on CaCl_2_ concentration but absence of CaCl_2_ dramatically reduces its formation.

Film formation occurred at all fibrinogen concentrations tested (0.05, 0.5, 1, 2.5 mg/ml; Figure 2I and Supplemental Figure 3D), even when the fibrinogen levels were not sufficient to form a clot that filled the whole volume of the liquid (Supplemental Figure 3E). Interestingly, as fibrinogen levels increased up to 1 mg/ml, MSF and film thickness decreased in a stepwise manner, signifying a decrease in film formation. Increases in fibrinogen concentration lead to shorter lag times and faster clotting rates (9). So in a trend similar to that for thrombin concentrations, a shorter lag time led to less fibrin accumulating at the air-liquid interface, leading to thinner films.

We also investigated film formation in samples from patients with dysfibrinogenemia and afibrinogenemia. To prevent changes in the clot or film structure with the addition of normal fluorescently labeled fibrinogen, we fluorescently labeled a non-antibody-binding protein (Affimer) (10) specific to fibrinogen with Alexa Fluor 488. This was added to the patient plasma before clotting, where it bound to any fibrinogen within the plasma. A clot was then formed and imaged using LSCM (Supplemental Figure 3F). Samples from patients with dysfibrinogenemia formed a film that had slightly higher MSF and thickness than clots formed from normal pool plasma. Afibrinogenemia patient samples did not form a fibrin clot or film, as expected (Figure 2J).

Films were similar in clots produced with reptilase (2.4 U/ml), which only cleaves fibrinopeptide A (and not fibrinopeptide B) from fibrinogen, and thrombin (Figure 2K and Supplemental Figure 3G). This demonstrates that film formation occurs during fibrinogen-to-fibrin conversion, regardless of the activating protease and independent of fibrinopeptide B (FpB) release.

We also assessed the effect of temperature on film formation (21°C room temperature, 31.5°C skin temperature, 37°C core body temperature). Although films formed at all 3 temperatures, film formation increased at least 20%–30% at 31.5°C compared with room temperature or core body temperature (Figure 2L and Supplemental Figure 3H). Enhanced film formation at skin temperature is consistent with a physiological role for fibrin films in cutaneous wounds and dermal injuries.

There were no differences in film MSF or thickness with or without factor XIII (FXIII) (3.7 μg/ml; Figure 2M and Supplemental Figure 3I), showing that FXIII is not necessary for film formation.

The role of platelets in film formation was investigated by comparing film formation in PPP and platelet-rich plasma (PRP). The lack of difference in MSF and film thickness between PPP and PRP indicates that platelets do not influence film formation (Figure 2N and Supplemental Figure 3J).

To investigate whether fibrinogen structural elements are essential for film formation, we tested natural and recombinant fibrinogen variants: γA/γA, the common fibrinogen isoform; γA/γ′, a heterodimeric splice variant that adds negative charge to the C-terminus of the γ-chain (11); γ3x, a triple γ-chain mutant that eliminates all γ-chain crosslinking (12); α220, in which the α-chain is truncated after residue Ser220, removing the connector and C-terminal globular domain of the αC-region; and α390, in which the α-chain is truncated after residue Asp390, removing the C-terminal globular domain. Clots formed with each of these variants produced fibrin films (Figure 2, O–Q, and Supplemental Figure 4, A–C). Differences in MSF were attributable to differences in polymerization rates and fibrin clot structure (13). These data indicate that neither the C-terminal domain of the γ-chain, the α-chain C-terminal connector region, nor the α-chain C-terminal globular domain is required for film formation.

Collectively, these findings show that fibrin produces a film that covers the blood clot at the air-liquid interface, and although other factors modulate film thickness or the amount of fibrin in the film, only fibrin is required.

### Mechanism of film formation.

**Figure 3:**
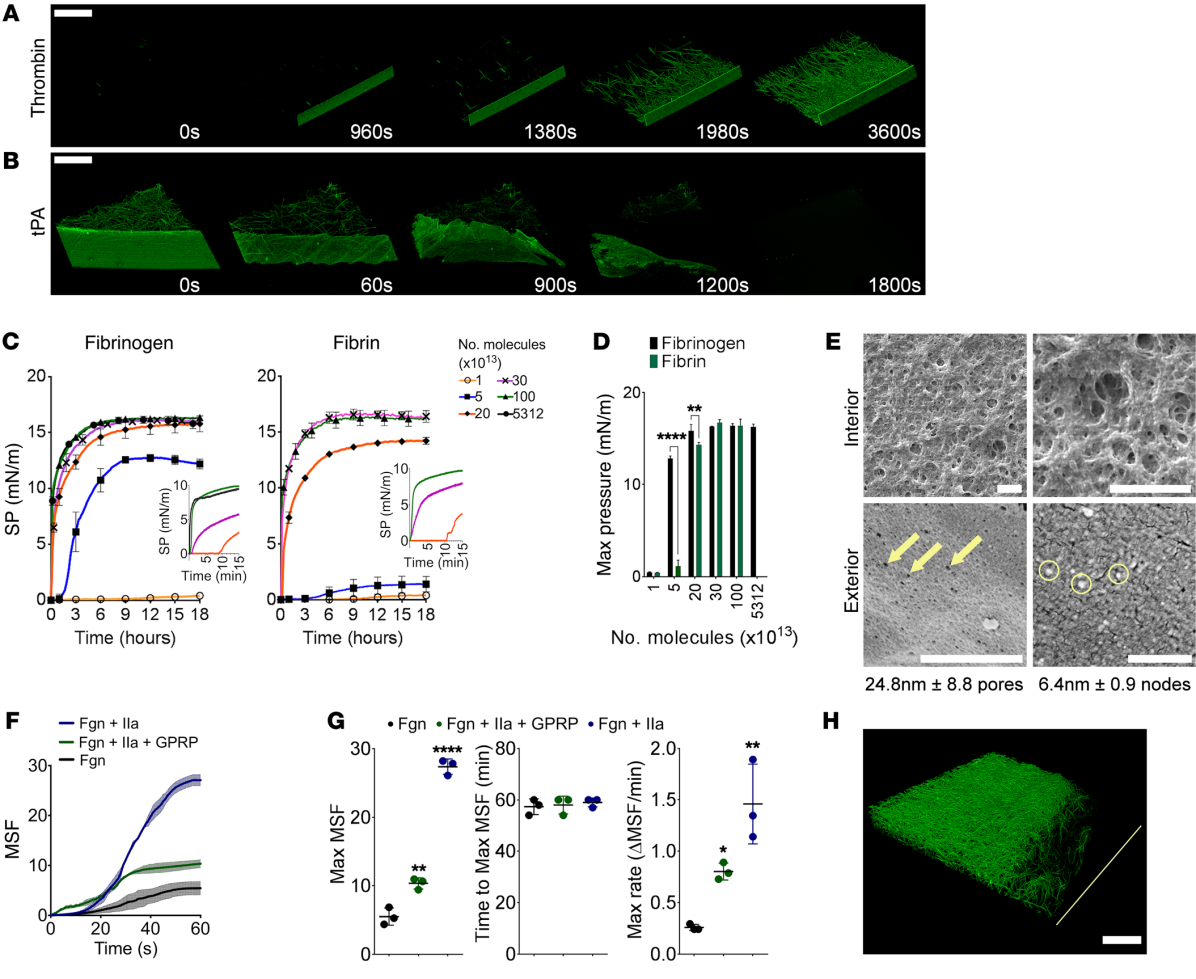
Mechanism of fibrin film formation. (**A**) Stills from videos of fibrin film formation over time in plasma when clotting was initiated with thrombin (*n* = 3 experiments). Scale bar: 50 μm. (**B**) Stills from videos of film lysis by tPA in plasma (*n* = 3 experiments). Scale bars: 50 μm. (**C**) Changes in surface pressure (SP) in the Langmuir-Blodgett trough with different fibrinogen or fibrin concentrations over time. Insets show early time points (0–15 minutes). Data are shown as mean ± SD, *n* = 3 experiments. SP, surface pressure. (**D**) Maximum Langmuir-Blodgett trough surface pressure of fibrinogen and fibrin with increasing concentrations. Data are shown as mean ± SD; **P < 0.01, ****P < 0.0001; *n* = 3 experiments; 2-way ANOVA, *F* = 293.5, degrees of freedom [df] = 1, *P* < 0.0001. (**E**) SEM images of the interior and exterior surfaces of films peeled away from plasma clots. Note differences in scale bars: interior, 3 μm; exterior, left 500 nm, right 50 nm. (**F**) Accumulation of MSF of clots from purified fibrinogen over time analyzed by LSCM. Data are shown as mean ± SD. (**G**) Maximum MSF, *n* = 3 experiments, *F* = 330.7, df = 2, *P* < 0.0001; time to maximum MSF, *n* = 3 experiments, *F* = 0.1875, df = 2, *P* = 0.8337; maximum rate of MSF increase, *n* = 3 experiments, *F* = 20.55, df = 2, *P* = 0.0021 as analyzed by LSCM. Data are shown as mean ± SD; *P < 0.05, **P < 0.01, ****P < 0.0001 compared with fibrinogen. (**H**) LSCM image of the air-liquid interface of a plasma clot formed in the presence of Tween-20 (0.1%), eliminating the film covering the clot. Yellow line represents the location of the air-liquid interface. Image is representative of *n* = 3 experiments. Scale bar: 50 μm. IIa, thrombin.

The transition of film to fibers (Figure 2E) and connection of film to the clot network via tethering fibers (Figure 2F) indicated that a specific biophysical mechanism governs fibrin film formation. To identify this mechanism, we used LSCM to visualize film formation and breakdown in plasma and purified fibrinogen solutions. Clot formation was initiated with thrombin (0.5 U/ml) in plasma (Figure 3A and Supplemental Video 1) or purified fibrinogen (Supplemental Figure 4D and Supplemental Video 2), and with tissue factor (5 pM) in plasma (Supplemental Figure 4E and Supplemental Video 3). Fibrin accumulated at the surface, forming a film before — or at approximately the same rate as — the fibrin fibers underneath the film that constitute the clot network. Once the clot was fully formed, breakdown was initiated with tissue plasminogen activator (tPA; 85 ng/ml) in plasma clots, or tPA plus plasminogen (25 μg/ml) in purified systems, and observed over time (plasma, Figure 3B and Supplemental Video 4; purified, Supplemental Figure 4F and Supplemental Video 5). In both cases, the films lysed at approximately the same rate as the network of fibrin fibers.

We next investigated accumulation of fibrinogen and fibrin at the air-liquid interface using a Langmuir-Blodgett trough, which analyzes monolayer film formation of amphiphilic molecules at the air-liquid interface (14, 15). Few previous studies have investigated fibrinogen at the air-liquid interface (16–19), and to our knowledge none have examined fibrin. Purified fibrinogen or fibrin monomers (fibrinogen with or without 1 U/ml thrombin and 5 mM Gly-Pro-Arg-Pro peptide [GPRP]) were injected into the subphase of the trough at increasing quantities (1 × 10^13^ to 5,312 × 10^13^) molecules of fibrinogen/fibrin), and surface pressure measurements were recorded over time. As protein accumulated at the surface over time, surface pressure increased and then at a maximum pressure (Figure 3C). At 1 × 10^13^ molecules, neither fibrin nor fibrinogen covered the surface, so there was no increase in surface pressure. At 5 × 10^13^ and 20 × 10^13^ molecules of fibrinogen/fibrin, there was a delay before fibrinogen or fibrin resulted in an increase in surface pressure. But greater quantities (30 × 10^13^ and 100 × 10^13^ molecules) of fibrinogen and fibrin caused an increase in surface pressure almost instantaneously (Figure 3C, insets, and Supplemental Table 2). Maximum surface pressure escalated with increasing amounts of fibrinogen and fibrin (Figure 3, C and D, and Supplemental Table 3). Fibrin resulted in significantly lower surface pressure levels than fibrinogen at 5 × 10^13^ and 20 × 10^13^ molecules, but as the quantity of fibrinogen/fibrin increased, these differences were no longer seen. The addition of more than 30 × 10^13^ molecules of fibrinogen did not further increase maximum pressure (100 × 10^13^ and 5,312 × 10^13^ molecules [1 mg/ml]), indicating that 30 × 10^13^ (0.006 mg/ml) is the maximum amount of fibrin/fibrinogen that can be accommodated at the surface. This was much lower than physiological fibrinogen levels (2–4 mg/ml). From this we calculated that this would allow for approximately 34 nm^2^ per fibrin/fibrinogen molecule. Assuming a fibrin(ogen) D-region is approximately 6.5–6.7 nm and the E-region is approximately 5–5.3 nm in diameter (20, 21), the half-staggered fibrin model (22) would allow for approximately 31–34 nm^2^ per fibrin/fibrinogen molecule, in close agreement with our experimental data. This indicates that fibrin/fibrinogen molecules are tightly packed and are positioned perpendicularly to the air-liquid interface.

In order to investigate the morphology of the film below the surface, we compared the interior and the exterior surfaces using SEM. The film was peeled away from the exterior of the clot and stretched over a coverslip with either the interior or exterior surface exposed (Figure 3E). The exterior surface was smooth, sometimes with very small pores (~24.8 ± 8.8 nm pores, yellow arrows) or with small nodes (~6.4 ± 0.9 nm domains, yellow circles). In contrast, the interior surface was rough, with holes, pores, and a fibrous structure, more closely resembling the clot network. In agreement with the Langmuir-Blodgett trough data, these data imply that fibrin films have an ordered, dense structure at the surface but become less ordered deeper in the film, where they transition into more fibrous structures.

With the interior surface looking similar to a clot network, we hypothesized that fibrin polymerization was involved in film formation. To assess this, we followed the accumulation of fibrin/fibrinogen below the surface and analyzed the effects of polymerization over time using LSCM. Time courses of polymerizing fibrin, fibrin monomers, or fibrinogen alone spiked with Alexa Fluor 488–fibrinogen were followed in a purified (1 mg/ml fibrinogen/fibrin) or plasma system (2× dilution). Accumulation was quantified by MSF (Figure 3, F and G). Purified fibrinogen accumulated at the air-liquid surface with a maximum rate of 0.26 ± 0.03 MSF/min, reaching maximum fluorescence (5.5 ± 1.3 MSF) after 57.3 ± 2.1 minutes. Fibrin monomers had a maximum rate approximately 3-fold faster than that of fibrinogen (0.8 ± 0.8 MSF/min), with twice as much protein accumulating at the surface (maximum MSF, 10.4 ± 0.8) after 58 ± 1.5 minutes. Polymerizing fibrin accumulated at the surface at the greatest rate, with a maximum increase in fluorescence of 1.46 ± 0.39/min, more than 5-fold the rate of fibrinogen. It reached a maximum fluorescence (27.1 ± 1.1 MSF) after 59 ± 0.7 minutes (Figure 3, F and G, Supplemental Figure 5A, Supplemental Table 4, and Supplemental Videos 2, 6, and 7). The same pattern was seen in plasma, with polymerizing fibrin accumulating at the surface more quickly than fibrin monomers and fibrinogen (Supplemental Figure 5, B–D, Supplemental Table 4, and Supplemental Videos 1, 8, and 9). This indicates that fibrin polymerization contributes to film formation by enhancing the accumulation of fibrin at the air-liquid interface. This effect is likely due to a combination of stronger interactions between the molecules of fibrin versus fibrinogen and an increase in hydrophobicity of fibrin (1, 23), which retains fibrin at the surface.

We next investigated film strength using atomic force microscopy (AFM). Young’s modulus was calculated for plasma samples with and without transglutaminase inhibitor T101 by fitting a Sneddon model for conical tips to all force curves found over the entire area that was imaged (Supplemental Figure 6, A and B). Fibrin fibers were visible under the film surface, and the Young’s modulus for these areas indicated greater stiffness than in places suspended between fibers. To find the stiffness of the film alone, we determined Young’s modulus by measuring areas between the underlying fibers of clots formed from plasma. The average Young’s modulus of the film was approximately 3-fold lower than in regions supported by fibers (suspended, 7.65 ± 0.24 kPa vs. fiber, 21.79 ± 2.79 kPa; *P* = 0.0001). Inhibition of cross-linking decreased the modulus of the suspended film further by just over 10-fold to 0.72 ± 0.14 kPa, which is slightly more than the decrease in fibrin stiffness previously reported in whole clots and single fibers (2- to 8.5-fold) (24–27). Areas supported by fibrin fibers decreased just over 4-fold to 5.1 ± 0.75 kPa in the absence of cross-linking (Supplemental Figure 6C). As a consequence, although fibrin crosslinking was not required for film formation (Figure 2L), it increased film elastic modulus.

Given that fibrin accumulates at the air-liquid interface, we hypothesized that film formation should be preventable by blocking the air interface with surfactant molecules. Accordingly, addition of Tween-20 (0.1%) prevented film formation and resulted in a dense, partly collapsed clot, probably due to the loss of surface tension (Figure 3H). Furthermore, blocking the air-liquid interface using mineral oil or petroleum jelly also prevented film formation (Supplemental Figure 6D).

**Figure 4:**
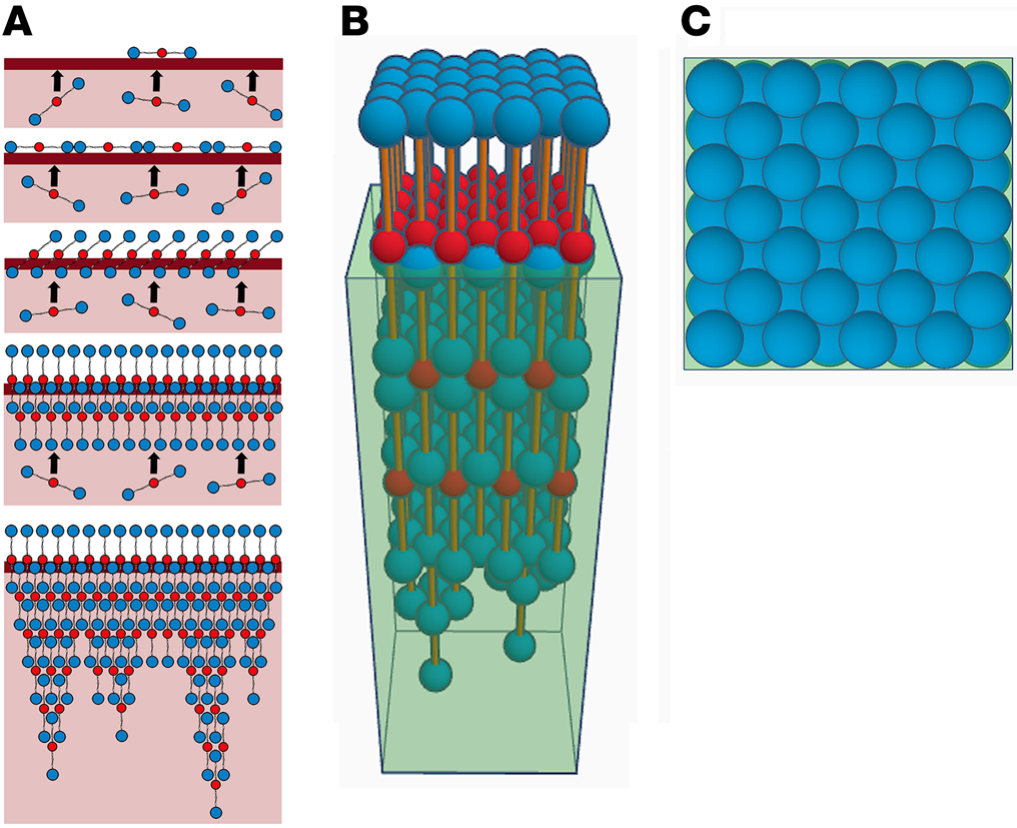
A model of fibrin film forming at the clot surface. (**A**) Proposed 2D model of fibrin(ogen) rising to the air-liquid interface and accumulating into a multilayer film with half-staggered fibrin molecules and tethering protofibrils (beginning of fibrin fibers). (**B**) Side-on view of a 3D model of the fibrin film with half-staggered fibrin molecules. D-regions are represented by 6.7-nm-diameter blue spheres; E-regions are represented by 5.3-nm diameter red spheres; and the connecting α-, β-, and γ-chains are represented by orange cylinders. The translucent box represents the liquid at the air-liquid interface (**C**) Top view of the 3D fibrin film model.

Based on the Langmuir (Figure 3, C and D) and accumulation data (Figure 3, F and G), we propose a model (Figure 4A) whereby, upon injury and exposure to air, fibrinogen molecules in the blood are rapidly adsorbed to the air-exposed surface, forming an organized monolayer at the air-liquid interface. Simultaneously, tissue factor stimulates fibrinogen cleavage via thrombin, leading to fibrin formation. Fibrin(ogen) continues to rise and accumulate at the air-liquid interface, packing in more molecules. At the same time fibrin begins to fit together in a half-staggered formation, allowing for the buildup of multiple layers of fibrin through A-a knob-hole interactions and the formation of tethering fibers (Figure 4, A–C). This leads to production of a dense layer of fibrin across the surface of the clot.

### Protection against microbes.

**Figure 5:**
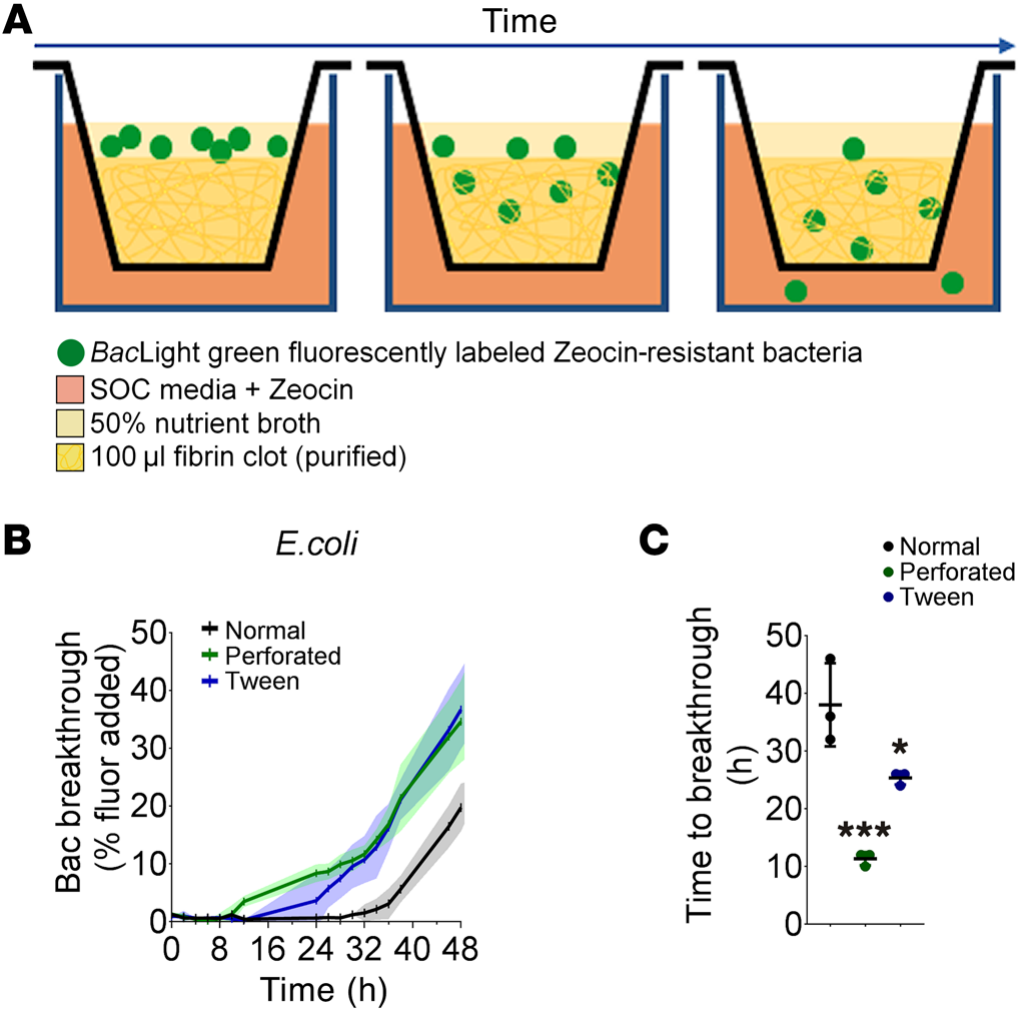
Fibrin film slows bacteria movement through clot. (**A**) Diagrammatic representation of the bacteria clot migration assay in the Boyden chamber. Bacteria moved from the 50% nutrient broth through the fibrin film (if present), into the fibrin clot, and then into the superoptimal broth with 20 mM glucose (SOC) medium. Quantity of bacteria moving through the clot over time was analyzed by fluorescence in the SOC media. (**B**) Movement of fluorescently labeled *E. coli* bacteria through the clots (displayed as quantity of fluorescent bacteria breaking through the clot as a percentage of fluorescent bacteria added) with 3 different film conditions: normal, perforated, and removal with Tween-20. Bac, bacteria. (**C**) Time taken for the first fluorescently labeled *E*. *coli* bacteria to break through the clot. *P < 0.05, ***P < 0.001 compared with normal clot; *n* = 3 experiments; 1-way ANOVA, *F* = 29.29, df = 2, *P* = 0.0008.

In view of the rapid formation of the fibrin film after exposure to an air interface, we hypothesized that an important physiological role of the film is to form an immediate barrier to protect the vascular breach against microbial invasion until white blood cells are recruited to the wound site (28). To investigate this hypothesis, we used a Boyden chamber to analyze migration of 3 types of bacteria commonly found in the natural skin flora (*Escherichia coli*, *Staphylococcus epidermidis*, and *Staphylococcus aureus*) through clots (Figure 5A). Bacteria were grown to 3.6 × 10^8^ to 8.5 × 10^8^ cells/ml, fluorescently labeled with *Bac*Light green, and applied to the external surface of 3 types of clots: (a) clots formed with 0.1% Tween-20 to prevent film formation, (b) clots where the surface was perforated with a needle to make holes in the film, and (c) normal clots with intact film. Fluorescent bacteria that moved through the clots were quantified over 48 hours (Figure 5B and Supplemental Figure 7A). The time taken for the first bacteria to break through the clots was defined as when fluorescence became greater than 2% of the total added fluorescent bacteria. No fluorescent bacteria migrated through the clots within the first 8 hours. *E*. *coli* migrated through the perforated (11.3 ± 1.2 hours) and Tween-20–treated clots (25.3 ± 1.2 hours) significantly faster than normal clots (38.0 ± 7.2 hours) (Figure 5C; *P* = 0.0008). A similar trend was seen with the 2 other bacteria (Supplemental Figure 7B). After the initial breakthrough, the rate of bacteria migration was comparable among clot types. This suggests the film prevents bacterial infiltration into the site of injury for at least 12–27 hours, allowing time for white blood cell recruitment and the underlying clot to fully form.

### Fibrin film formation in vivo and blood cell retention.

**Figure 6:**
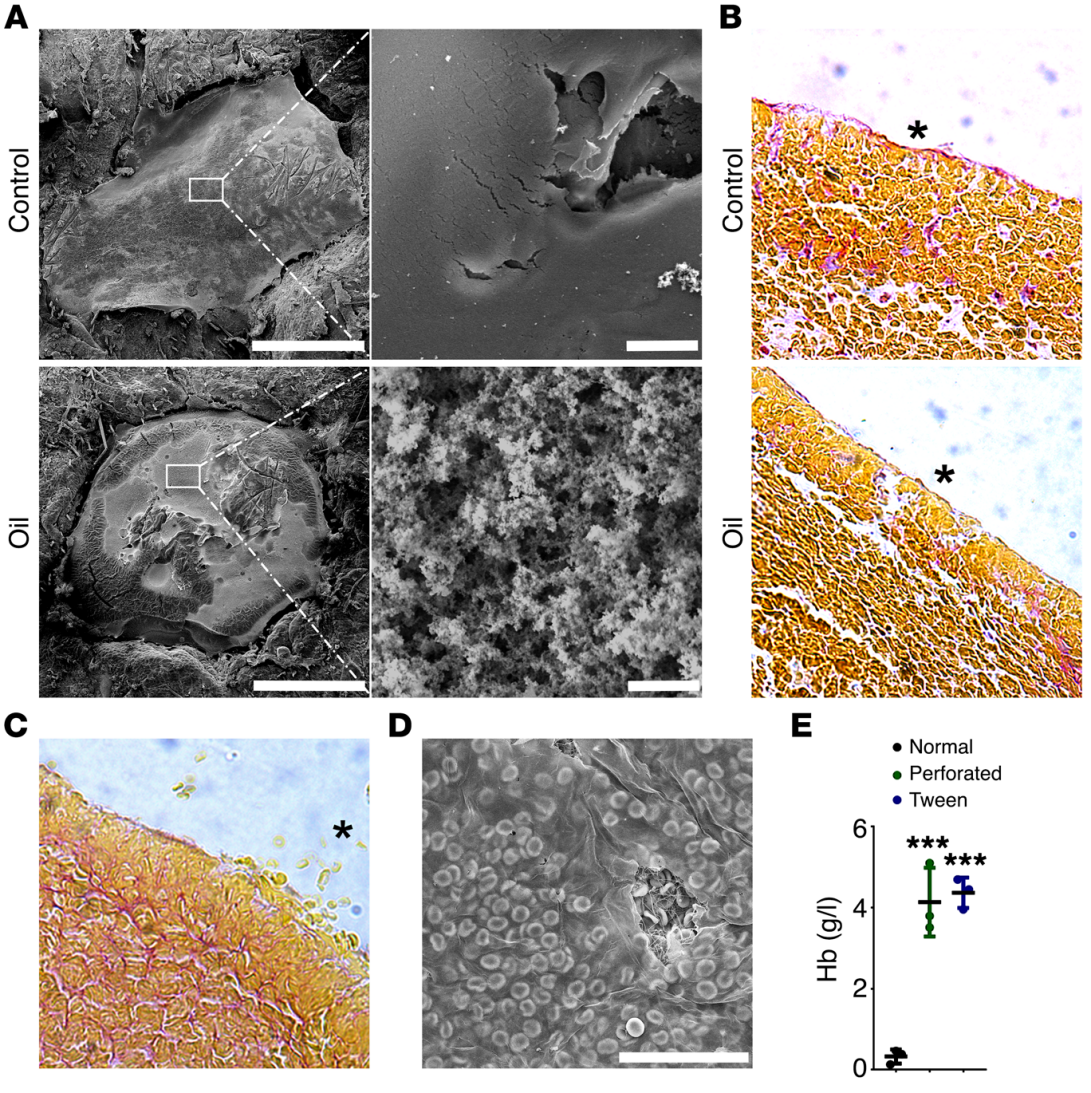
Fibrin film formation in vivo. (**A**) Clots were formed in a ventral, dermal puncture injury model in mice and left clear or covered with a layer of mineral oil, fixed, dehydrated, and imaged by SEM. The white box represents the area of magnification for the image to the right. Clots covered with oil showed a rough fibrous clot surface, and clots left untreated showed a smooth fibrin film covering the clot. Images are representative of *n* = 4 mice. Scale bars: left, 1 mm; right, 2 μm. (**B**) Clots left clear or with a layer of oil covering the surface from the dermal puncture model were surgically removed, and cross sections were stained with MSB (erythrocytes in yellow, fibrin in pink). Fibrin film shows as continuous pink layer; clot appears yellow interspersed with pink. Asterisks highlight the air-liquid interface. Images are representative of *n* = 4 mice. (**C**) Clots from the murine dermal puncture model covered with oil showed extrusion of red blood cells from the clot in the absence of a fibrin film. Asterisk highlights extrusion of red blood cells from the clot. Image is representative of *n* = 4 mice. (**D**) Clots produced with human whole blood and thrombin and imaged by SEM demonstrated containment red blood cells by the fibrin film. Image is representative of *n* = 3 individuals. Scale bar: 40 μm. (**E**) Hemoglobin retention assay in normal, perforated, and Tween-20–treated clots. ***P < 0.001 compared with normal clot; *n* = 3 individuals; 1-way ANOVA, *F* = 52.14, df = 2, *P* = 0.0002. Hb, hemoglobin.

To investigate the role of the film on bacteria migration in vivo, we established a murine dermal injury model. Initially, we investigated whether fibrin film formation occurred ex vivo in mouse blood. Clots were formed with whole blood from WT or fibrinogen-deficient mice and were prepared for SEM. Blood from WT mice formed a fibrin film at the air-liquid interface, whereas blood from fibrinogen-deficient mice did not (Supplemental Figure 8A). We next established a murine dermal injury model in which a 2-mm puncture was created in the skin on the back or abdomen of anesthetized mice. The injury was either left uncovered to allow clotting exposed to the air or immediately covered with a thin layer of oil to remove the air-liquid interface, which was washed off with saline once the clot had formed. After 60 minutes, clots and surrounding skin were surgically removed, processed, and imaged by SEM (Figure 6A). In the untreated control section, a film was present on the exterior surface of clots. Consistent with the in vitro experiments, oil treatment prevented film formation, leaving the fibrous fibrin network exposed and clearly visible by SEM (Figure 6A). Some of these clots were embedded in paraffin, sectioned, and stained using Martius, Scarlet, and Blue (MSB) or were probed with a fibrin-specific antibody (59D8). In the untreated control sections, the film can be seen along the surface of the clot, as a thin pink layer with MSB (Figure 6B) or a thin brown layer with 59D8, confirming that fibrin makes up the film in vivo (Supplemental Figure 8B). In clots treated with oil, the film was not present. This showed that the oil prevented film formation in vivo and could be used to assess bacterial migration and proliferation in clots without a film.

During the establishment of our in vivo model, we found histology sections of clots formed in the presence of oil — and therefore lacking the film — showing regions of loose cells that were not retained within the clot (Figure 6C). In micrographs of the control clots, we found erythrocytes pushed against the inside of the film (Figure 6D). These findings suggested that the fibrin film helps to prevent leakage of blood cells from dermal lesions. To investigate this, we formed clots from whole blood with no intervention, in the presence of Tween-20 (0.1%), or with the film being perforated after 1 hour. After 2 hours the amount of hemoglobin released from the clot was measured. We found that the quantity of hemoglobin released was more than 12-fold greater in the Tween-20–treated (4.37 ± 0.38 g/l) and perforated clots (4.14 ± 0.85 g/l), with almost no hemoglobin being released from the normal clot (0.32 ± 0.18 g/l, *P* = 0.0002; Figure 6E). These data show that the film plays a role in the retention of red blood cells within the clot.

### Protective role of fibrin film in a murine dermal injury model.

**Figure 7:**
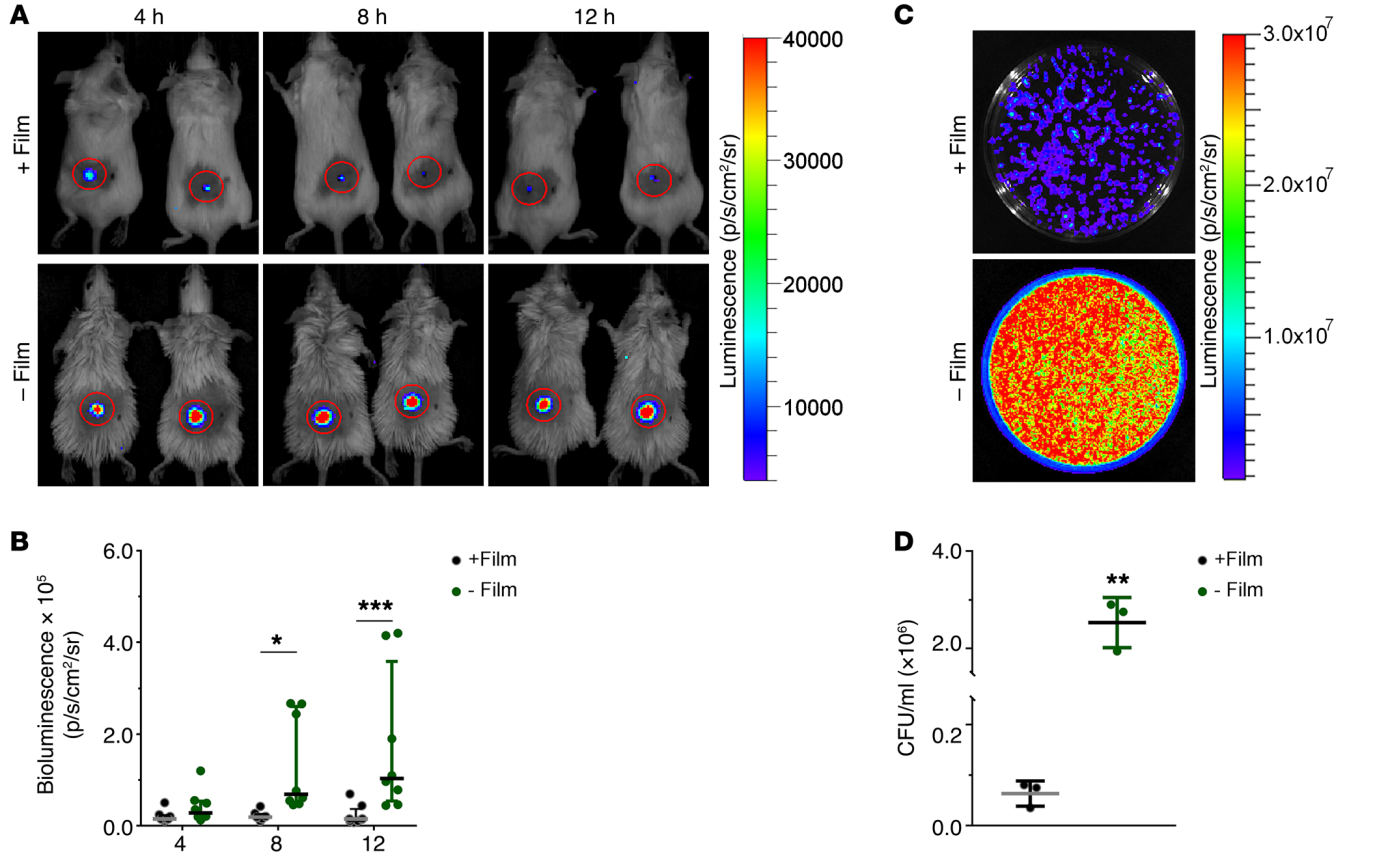
Fibrin film slows bacteria proliferation and dissemination in vivo. (**A**) Measurement of bioluminescent bacteria proliferation with or without film in a murine dermal injury model over time. Images are representative of *n* = 8 mice (**B**) Quantification of bioluminescent bacteria in this model. *P < 0.05, ***P < 0.001, *n* = 8 mice, Kruskal-Wallis test, Kruskal-Wallis statistic = 36.55, 6 groups, *P* < 0.0001. (**C**) Measurement of bioluminescent bacteria from wound and surrounding skin with and without film after 12-hour spread on agar plates. Images are representative of *n* = 3 mice. (**D**) CFU/ml of bacteria from wound and surrounding skin with or without film after 12 hours. **P < 0.01, *n* = 3 mice, unpaired *t* test, *t* = 8.26, df = 4, *P* = 0.0012.

We next investigated the role of the fibrin film in protection against bacterial proliferation and dissemination in vivo. After the clots were formed as described above (with or without a layer of oil), 2 μl bioluminescent *Pseudomonas aeruginosa* (4 × 10^5^ CFU), a Gram-negative flagellated bacterium associated with severe hospital-acquired infections and significant resistance to antibiotics, were deposited on the surface of the clots covering the dermal punctures. Mice were imaged every 4 hours using a 3D Ivis Spectrum In Vivo Imaging System to assess bacterial proliferation. Control experiments were run in parallel to demonstrate oil had no effect on bacterial proliferation. In the mice with an intact fibrin film, the bioluminescence did not increase over 12 hours, showing that the bacteria did not proliferate over this period in these mice (Figure 7, A and B, and Supplemental Table 5). In contrast, in mice without a fibrin film the bioluminescence was significantly higher after 4 hours and increased over time up to 1.034 × 10^5^ p/s/cm^2^/sr (interquartile range [IQR], 0.470, 0.359; where “p” is photons and “sr” is steradians), compared with 0.151 × 10^5^ p/s/cm^2^/sr (IQR, 0.107, 0.371; *P* = 0.0005) in the mice with films (Figure 7, A and B, and Supplemental Table 5). These data show that the bacteria were proliferating within the wound in the absence of the film. To confirm the difference in bacteria numbers, we harvested and mechanically homogenized the clots and surrounding skin from sacrificed animals after 12 hours. Serial dilutions of the homogenates were plated on agar for analysis of bioluminescence and bacterial CFU (Figure 7, C and D). In agreement with the bioluminescence data, the mice that had no film on their clot showed a much greater number of bacteria (2.53 × 10^6^ ± 0.298 × 10^6^ CFU/ml) within the wound and skin than the mice that had an intact clot film (0.064 × 10^6^ ± 0.014 × 10^6^ CFU/ml). These data demonstrate that the film has a protective role, slowing or preventing the proliferation of bacteria and reducing the movement of bacteria into wounds and skin in the first 12 hours after injury.

## Discussion

This study demonstrates that fibrin plays an unexpected role in hemostasis, producing a film that not only encapsulates the clot and thereby retains cells, but also functions as a protective layer at the interface between the clotting blood and the air, preventing microbial infection. The structural characteristics of this fibrin film are completely different from those of the fibrin fiber network. The film is composed of a thin continuous layer of fibrin and is connected to the fibrous fibrin network through tethering fibers. Film formation is initiated by the exposure of blood to the air interface and accumulates due to the conversion of fibrinogen to fibrin, and the resultant increase in hydrophobicity of the fibrin molecule (1, 23). The unique fibrin polymerization mechanisms, which involve knob-hole interactions, lead to fibrin being able to produce both films and fibers (29), and we show that this culminates in a remarkable integrated clot structure that includes a fibrin film covering a network of fibers. This finding transforms our understanding of blood clots at injury sites and sheds new light on a major enigma — how the clot ends at the air-blood interface — that has troubled the field for a long time.

The film has previously been observed in our laboratory and those of others in SEM images of blood, plasma, or fibrin clots. However, until now, it has been overlooked, as it was alleged to be an artefact of sample preparation. The close association of fibrin clot structure with thrombosis risk (3, 30, 31) has led researchers to focus on areas of the clot not covered by the fibrin film, i.e., away from the clot-air interface, or in areas where the film may have ruptured during sample processing. Previous studies have shown that fibrin is able to produce sheet-like structures, across gaps in a striated substrate, for example (29), or in blood clots (32). However, the physiological relevance of these structures and the mechanism of formation were not explored. Here, we investigated the film in detail and found it to be a physiological product of clotting and fibrin formation.

Fibrinogen and fibrin have previously been associated with protection against systemic infection (33, 34). Our new data demonstrate that fibrin additionally plays an important role in controlling local infection at injury sites, forming an instant barrier that slows the proliferation of bacteria and delays its movement into the wound, allowing the underlying clot to fully form and create a more permanent barrier. Lack of this clot barrier film is expected to lead to a significant increase in infection. Our data indicate the need for future studies on the application of petroleum jelly to help stem bleeds from injuries in contact sports or to a wound after minor surgery. Another area of clinical interest is afibrinogenemia, since the lack of fibrin film formation in this disease may lead to increased risk of persistent infections from minor dermal lesions (Supplemental Figure 9). As a corollary, an engineered fibrin film would be expected to improve recovery from minor and major injuries and reduce the probability of severe infection.

Our findings of the extraordinary formation of a protective fibrin film on the blood clot exterior reveal a mechanism in hemostasis that helps retain blood cells and control microbial infection. Clot film formation appears an important physiological process that should be exploited in the future to improve recovery and healing from minor and major injuries.

## Methods

### Materials.

Human plasma fibrinogen, plasminogen-depleted (Calbiochem), was further purified by immunoaffinity chromatography (IF-1 mAb, 10 mg; Kamiya Biomedical) as previously described (35) to eliminate FXIII (36). Alexa Fluor 488– or Alexa Fluor 594–fibrinogen (Invitrogen) was reconstituted to 2.5 mg/ml, human thrombin (Calbiochem) was reconstituted to 250 U/ml; and tPA (Pathway Diagnostics) and Glu-plasminogen (Enzyme Research Laboratories) were diluted in 0.05 M Tris-Base, 0.1 M NaCl, pH 7.4 (TBS) to 1 mg/ml and 990 μg/ml, respectively, and stored at –80°C. Human FXIII-A_2_B_2_ was isolated from contaminating albumin and glucose from Fibrogammin P (Zedira) by Sepharose-6B gel filtration as described previously (12). FXIII was diluted in TBS to 110 μg/ml and stored at –80°C. All other chemicals were obtained from Sigma-Aldrich unless stated otherwise.

### Whole blood.

Samples of free-flowing blood were collected from the antecubital vein of healthy volunteers. Blood was collected on 0.109 M sodium citrate and was used within 4 hours.

### PPP and PRP.

Free-flowing blood was obtained from the antecubital vein of 24 healthy volunteers as described above. The blood was centrifuged at 2,400 *g* for 20 minutes for PPP or at 200 *g* for 10 minutes for PRP. PPP and PRP from 6 individuals were used to form clots for film formation comparison. PPP samples from 24 volunteers were pooled, aliquoted, and snap-frozen in liquid nitrogen and stored at –80˚C.

### Purification of γA/γA and γA/γ′ fibrinogen.

γA/γA and γA/γ*′* fibrinogens were isolated as previously described (8, 37, 38). In short, the variants were separated using a DE-52 column on an ÄKTA Avant 25 (GE Healthcare). Fibrinogen was dissolved in 39 mM Tris, 65 mM H_3_PO_4_, 0.5 mM PMSF, 1 mM benzamidine, and 5 mM ε-aminocaproic acid, pH 8.6. Samples were eluted using a concave gradient from 0% to 100% over 13 column volumes (with increments of 5% over the first 6 and increments of 10% over the next 7 column volumes) of 500 mM Tris, 650 mM H_3_PO_4_, 0.5 mM PMSF, 1 mM benzamidine, and 5 mM ε-aminocaproic acid, pH 4.2. The fibrinogens were concentrated and dialyzed in 50 mM Tris-HCl and 100 mM NaCl pH 7.4. The purity of the γA/γA and γA/γ′ preparations was checked on a NuPAGE unit with 4%–12% Bis-Tris gradient gels (Invitrogen), and aliquots were stored at –80°C.

### Mutant fibrinogen expression.

Recombinant human fibrinogen expression and purification have been described previously (12, 35). Briefly, truncations (αSer220 and αAsp390) and γ-chain mutations (Q398N/Q399N/K406R, referred to as γ3x) were established through the use of a QuikChange II Site-Directed Mutagenesis Kit (Agilent Technologies). The expression vector pMLP encoded the entire cDNA for either the α- or γ-chain. Primers were designed to change residues at desired locations (γ398, γ399, γ406) or to create a stop codon at α221 and α391. Mutations and truncations were confirmed by sequencing (MRC Protein Phosphorylation and Ubiquitylation Unit [PPU] DNA Sequencing and Services, University of Dundee, Dundee, United Kingdom). Plasmids were transfected into CHO cells containing the remaining fibrinogen chains. A second plasmid was transfected for selection (pMSV-his). Recombinant fibrinogen WT, γ3x, and α-truncations were produced in roller bottles containing microcarrier beads and DMEM/F12 (Thermo Fisher Scientific), and medium was supplemented with aprotinin, 5 μg/ml insulin, 5 μg/ml transferrin, and 5 ng/ml sodium selenite (Roche). Medium was collected and replaced every 48 hours and stored at –40°C with the addition of 150 μM PMSF, and harvested for 8 weeks. The fibrinogen was precipitated overnight with 40% saturated ammonium sulfate (VWR International) and a mixture of protein inhibitors (20 mM MES hydrate pH 5.6, 5 mM 6-aminihexanoic acid, 5 mM benzamidine, 100 μM PMSF, 1 μM pepstatin, 1 μM leupeptin). The precipitated medium was centrifuged at 14,500 *g* for 45 minutes without brakes at 4°C in an Avanti J-265 XPI (Beckman Coulter). The pellet was resuspended in a protein cocktail (333 mM NaCl, 222 mM Tris, 111 μM PMSF, 5 μM pepstatin, 5 μM leupeptin, 1 mM EDTA, 11 U/ml trypsin inhibitor, 5 mM benzamidine, 5 mM 6-aminihexanoic acid), incubated for 30 minutes at 4°C, and centrifuged at 43,000 *g* for 30 minutes. Supernatant was collected and kept at –80°C. Samples were purified by immunoaffinity chromatography (IF-1 mAb, 10 mg; Kamiya Biomedical) as previously described (35). Fractions containing fibrinogen were pooled and stored at –80°C. Fibrinogen was concentrated and dialyzed in 50 mM Tris-HCl and 100 mM NaCl pH 7.4. Protein integrity was assessed by SDS-PAGE.

### SEM: whole blood, plasma, purified fibrinogen, thrombin/tissue factor.

Clots for SEM were prepared by adding 10 μl activation mixture (whole blood/plasma: 0.5 U/ml human thrombin or 5 pM tissue factor [Diagnostica Stago], CaCl_2_ 5 mM; purified fibrinogen: 0.5 U/ml human thrombin, 5 mM CaCl_2_ final concentrations, in TBS) to 100 μl of whole blood, plasma, or purified fibrinogens with or without FXIII (final concentrations: fibrinogen, 1 mg/ml; FXIII, 3.7 μg/ml). The clotting mixture was immediately transferred to pierced Eppendorf lids. Clots were left to form in a humidified chamber at room temperature for 2 hours. Clots were washed with saline solution to remove excess salt and prepared for microscopy by fixation in 2% glutaraldehyde solution for at least 120 minutes. Clots were further washed with sodium cacodylate buffer (67 mM C_2_H_6_AsNaO_2_, pH 7.4) and dehydrated in a series of increasing acetone concentrations (30%–100%). Clots were critical point dried with CO_2_, mounted onto stubs, and sputter coated with platinum using a Cressington 208 HR (Cressington Scientific Instruments). Each clot was formed in duplicate and imaged in 5 areas, at different magnifications (2,500×, 5,000×, 10,000×, 20,000×, 25,000×, and 50,000×) using a Hitachi SU8230 high-performance cold field emission (CFE) SEM (Chiyoda).

### LSCM; whole blood, plasma, and purified fibrinogen.

Reaction mixtures were prepared by diluting whole blood or plasma by half with saline or TBS, respectively, and spiked with 25 μg/ml Alexa Fluor 594– or Alexa Fluor 488–fibrinogen, respectively, and 5 mM CaCl_2_. Human thrombin (0.5 U/ml) or 5 pM tissue factor was added to initiate clotting. Purified fibrinogen (1 mg/ml) was spiked with 25 μg/ml Alexa Fluor 488–fibrinogen, with or without FXIII (3.7 μg/ml) and 5 mM CaCl_2_. 0.5 U/ml human thrombin was added to initiate clotting. Immediately after the initiation of clotting, a 30-μl drop of the mixture was transferred to the center of the well of an uncoated 8-well Ibidi slide (Ibidi GmbH), and the slide was transferred to a dark humidity chamber for 4 hours at room temperature. Imaging was performed using an inverted Zeiss LSM880 microscope with a 40× oil immersion objective lens. Fibrin clots were prepared in duplicate, 4 images were taken at the air-liquid interface for each clot (Supplemental Figure 10A), and *Z*-stacks (20 μm, 30 slices) were combined to form 3D images (ZEN 2.1 black, Zeiss).

### Conditions: thrombin, calcium, fibrinogen concentration, reptilase, temperature, platelets, fibrinogen variants.

LSCM and SEM were carried out as above, but with changes to some of the conditions. Experiments were carried out in which clotting was initiated with different thrombin (final concentrations: 0.1, 0.5, 1, 10 U/ml), CaCl_2_ (0, 5, 10, 20 mM), and fibrinogen concentrations (0.05, 0.5, 1, 2.5 mg/ml) in a purified system. Clotting was also initiated with reptilase (2.4 U/ml; Diagnostica Stago) to investigate the effects of only cleaving fibrinopeptide A compared with thrombin (0.5 U/ml).

The effects of temperature were investigated by incubating the reaction mix at different temperatures (21°C, 31.5°C, 37°C) before and throughout clotting. Average skin temperature was determined as 31.5°C using a contact thermometer on the forearm and hand. Immediately after clotting initiation, a 30-μl drop of the reaction mixture was transferred into the center of the well of an uncoated 8-well Ibidi slide, and the slide was transferred to a dark humidity chamber in an incubator at the appropriate temperature for 4 hours.

The effects of platelets on film formation were studied by comparing film formation in PPP and PRP from 6 healthy volunteers. Reaction mixtures were prepared by diluting PPP or PRP by half with saline and spiked with 25 μg/ml Alexa Fluor 594–fibrinogen and 5 mM CaCl_2_. Tissue factor (5 pM) was added to initiate clotting.

The effects of fibrinogen variants were investigated with purified γA/γA or γA/γ′, γ3x mutant, or α220 or α390 mutant (each at 2.94 μM).

### Fibrin(ogen)-binding Affimers for imaging the film in dys- and afibrinogenemia.

Free-flowing blood was obtained from the antecubital vein of 2 dysfibrinogenemia patients (FGA c.112A>G, p.R38G; FGG c.901C>T, p.R301C) and 3 afibrinogenemia patients (all with the FGA c.635T>G, p.L212X mutation) as described above. The blood was centrifuged at 2,400 *g* for 20 minutes for PPP. These samples were aliquoted, snap-frozen in liquid nitrogen, and stored at –80˚C. To image the plasma samples, a fibrin(ogen)-specific Affimer was isolated from an Affimer phage display library using a previously described screening process (10). This Affimer was fluorescently labeled using an Alexa Fluor 488 protein labeling kit (Invitrogen) according to manufacturer’s instructions. Fluorescently labeled Affimer was then added to normal pool plasma or patient plasma at 17.6 μM and incubated for 30 minutes. Clotting was initiated with 5 mM CaCl_2_ and 0.5 U/ml human thrombin, and a 30-μl drop of the mixture was immediately transferred to the center of the well of an uncoated 8-well Ibidi slide. Clots were incubated for 4 hours in a humidity chamber and were then imaged by LSCM as described above.

### Confocal time series formation/lysis.

To investigate film formation, the reaction mixture was produced as above for both plasma and purified fibrinogen. A 27-μl drop of this mixture was placed into the center of a well of an uncoated 8-well Ibidi slide. 2 μl thrombin (0.5 U/ml) or tissue factor (5 pM) was added to the drop, which was immediately observed by LSCM. The effects of preventing polymerization were investigated by preincubating fibrinogen (1 mg/ml) or plasma with GPRP (5 mM) for 20 minutes. Thrombin (3 μl, 0.5 U/ml) was added to the drop, which was immediately observed by LSCM using a 40× oil immersion objective lens with 29 × 0.7–μm *Z*-stacks every 60 seconds.

To investigate fibrinolysis, clots were formed as described above. After film formation, 5 μl tPA (85 ng/ml) was added to plasma clots, and 5 μl plasminogen (25 μg/ml) and tPA (85 ng/ml) was added to purified clots. The clot was immediately observed by LSCM. Formation and lysis were observed by LSCM using a 40× oil immersion objective lens. 29 × 0.7–μm *Z*-stacks were obtained every 60 seconds. 3D videos were created from the *Z*-stacks.

### Preventing film formation: oil, Tween-20, petroleum jelly.

The reaction mixture was made up for plasma as mentioned above for reactions with oil or petroleum jelly. For oil experiments, mineral oil was carefully placed around the drop to fill the confocal well before clotting, to eliminate the air-liquid interface before clotting was initiated. For petroleum jelly experiments, 27 μl of reaction mixture was injected into a small ball of petroleum jelly that had been placed in the middle of the well of a slide. For reactions with Tween-20, the reaction mix was incubated with 0.1% Tween-20 before clotting. Using a pipette, thrombin (0.5 U/ml) was added to the reaction mixture to initiate clotting. The slide was transferred to a humidity chamber for 4 hours at room temperature. Imaging was performed using an inverted Zeiss LSM880 microscope with a 40× oil immersion objective lens. Each fibrin clot was prepared in duplicate, 4 images were taken of each sample, and *Z*-stacks were combined to form 3D images.

### Film thickness.

The thickness of the film was measured on confocal images 60 times per image using ImageJ (version 2.0, NIH), and the average thickness for each image was used to compare among conditions.

### Fluorescence measurements.

To quantify film fluorescence, an outline was drawn around the film on each focal plane. Area, integrated density, and 3 adjacent background readings were made using ImageJ. Corrected film fluorescence was calculated as follows: integrated density – (area selected × mean fluorescence of background readings). This was calculated for each focal plane. The average of the corrected film fluorescence of each focal plane was taken and was divided by 10,000 arbitrarily to simplify the numbers, and was called MSF. To validate this method, measurements of film thickness from confocal images of 72 separate samples were taken. Sixty measurements of film thickness were taken per image using ImageJ, and the average thickness for each image was correlated with MSF taken from the same image (Supplemental Figure 10B).

### Film peel.

Clots were generated by spreading 100 μl plasma with or without 1,3,4,5-tetramethyl-2-[(2-oxopropyl)thio]imidazolium chloride (T101, FXIII inhibitor, 1 mM; Zedira) or purified fibrinogen (1 mg/ml) with or without FXIII (3.7 μg/ml) into an approximately 2 × 2–cm square on coverslips. 10 μl of activation mixture (human thrombin, 0.5 U/ml and CaCl_2_, 5 mM) was added to initiate clotting. After 4 hours a hypodermic needle was used to peel the film away from the surface of the clot (Figure 2A). The film was either reduced and run on SDS-PAGE or was stretched over a coverslip exposing either the interior surface or exterior surface of the film and was prepped for SEM.

### SDS-PAGE and Western blot analysis.

Clots were made using plasma by the addition of thrombin (0.5 U/ml) and CaCl_2_ (5 mM), with and without FXIII inhibitor T101 (1 mM). The fibrin film was removed from each clot and reduced by the addition of NuPAGE sample reducing agent (100 mM DTT) and heating at 95°C for 15 minutes. Fibrin samples were prepared by the formation of a clot with IF-1 fibrinogen (1 mg/ml), addition of thrombin and CaCl_2_, and reduction as described above. FXIII-, BSA-, and IF-1–purified fibrinogen samples were reduced in a similar way and run alongside the films to help identify bands in the gel, and as controls for the blots. Protein concentrations were determined using NanoDrop to load 2 μg of each protein sample on 2 identical 4%–12% NuPAGE Bis-Tris gels. After running, one gel was stained using GelCode Blue Safe Protein Stain (Thermo Fisher Scientific), and one was transferred to a PVDF membrane (Thermo Fisher Scientific). The membrane was blocked overnight using 4% skim milk in 50 mM Tris, 150 mM NaCl, 0.1% Tween-20. Polyclonal rabbit anti–human fibrinogen antibody (A0080; Dako) was added to the blot in blocking buffer and detected using goat anti-rabbit HRP secondary antibody (P0448; Dako). Signal was detected using SuperSignal West Pico Chemiluminescent Substrate (Thermo Fisher Scientific).

### AFM sample preparation.

Samples for AFM mechanical measurements and imaging were prepared from normal pool plasma with a 1:2 final dilution. Briefly, plasma was mixed with an activation mixture of CaCl_2_ (5 mM) and thrombin (0.5 U/ml) and placed onto a 34-mm-diameter tissue culture dish (TPP) with the plasma mixture covering a 10 × 10–mm square. For samples with T101, a final concentration of 1 mM was added to the mixture. Samples were then placed into a humidity chamber and allowed to clot for 1 hour. Samples were hydrated with 3–5 ml of 50-mM Tris, 100 mM NaCl, and placed on the AFM sample stage. Imaging and force measurements were performed on a JPK Instruments NanoWizard 4 and a Zeiss AxioObserver D1 in qi mode with 10-nm-radius AFM probes (CB3, qp-BioAC, Nanosensors). All measurements were done in triplicate over a 15 × 15–μm square.

### Langmuir-Blodgett trough.

Surface tension measurements were used to determine fibrinogen and fibrin adsorption at the air-liquid interface. Measurements were performed with an extra small KSV NIMA double-barrier Langmuir-Blodgett trough (203 × 50 × 1.2 mm) with surface pressure sensor, based on the Wilhelmy method, with a Whatman CHR1 chromatography paper plate (perimeter, 20.6 mm; accuracy in surface pressure, 0.01 μN/ml; KSV NIMA, Biolin Scientific). Because adsorption measurements are sensitive to the presence of impurities, extreme care was taken to ensure that all materials and instruments used in this study were clean. The trough and barriers were cleaned with methanol and rinsed with deionized water before each run. A new Wilhelmy plate was used for each run. Due to the duration of the experiment, the trough was maintained in a dust and draught exclusion cabinet throughout the measurements to minimize the presence of impurities from the atmosphere. A humid atmosphere was maintained by putting a trough of water in the enclosing box. The subphase was composed of 30 ml of 0.2 μm filtered TBS pH 7.4 at room temperature. The surface was checked prior to each measurement to ensure that it was clean by moving the barriers to the center and confirm that the surface pressure was below 0.3 mN/m. The system was set to record surface pressure every second for 18 hours. The desired protein concentration was achieved by diluting in TBS (fibrinogen, 1, 5, 20, 30, 100, 5,312 × 10^13^ molecules [final concentrations, 0.00018–1 mg/ml]; fibrin 1, 5, 20, 30, 100 × 10^13^ molecules [final concentrations, 0.00018–0.019 mg/ml]), and the sample was injected into the subphase in one 50-μl injection with a Hamilton Gastight syringe (Thermo Fisher Scientific) and left for 18 hours. Fibrin monomers were formed by preincubating fibrinogen with GPRP (5 mM) overnight, followed by incubation with thrombin (0.5 U/ml) for 2 hours. As a control a 50 μl mixture of thrombin (0.5 U/ml) and GPRP (5 mM) was run on the trough to show that it caused no change in surface pressure.

### Bacteria migration assay.

To investigate bacteria migration, an assay was set up using a Boyden chamber (VWR) as shown in Figure 5A. Three types of bacteria — *Escherichia coli* (ATCC 13706), *Staphylococcus epidermidis* (ATCC 12228), and *Staphylococcus aureus* (ATCC 29247) — commonly found in the natural skin flora were transformed with pSELECT-zeo plasmid (Invivogen) to provide resistance to antibiotic Zeocin. A single colony was picked for each bacterium and was grown up overnight (3.6 × 10^8^ to 8.5 × 10^8^ cells/ml) in nutrient broth containing Zeocin (25 μg/ml, Sigma-Aldrich). Three clots for each strain of bacteria were formed in a Boyden chamber (0.8-μm pores, Millipore), 2 normal (1 mg/ml fibrinogen, 5 mM CaCl_2_ , 0.5 U/ml thrombin) and 1 in the presence of Tween-20 (0.1%), and left overnight in a humidity chamber at room temperature. The next day the film on one of the normal clots for each bacterium was perforated by running a hypodermic needle across the surface of the clot. Each bacterium was fluorescently labeled with *Bac*Light green following the manufacturer’s instructions (Thermo Fisher Scientific). The bacteria were then spun down and resuspended in 50% nutrient broth to remove any unused label and were checked for fluorescence levels. Zeocin (25 μg/ml) was added to SOC media (Thermo Fisher Scientific), and 1 ml was added to each well of the plate. Each clot was placed in the well, and 300 μl of labeled bacteria was added in the chamber on top of each clot. SOC media (50 μl) was taken from each well and replaced with fresh SOC media every 2 hours for 48 hours and measured for fluorescence. The time to the first bacteria breaking through the clots was defined as when fluorescence reached a level greater than 2% of the added fluorescent bacteria.

### Ex vivo WT and fibrinogen-deficient mouse blood clots.

C57BL/6 and fibrinogen-deficient mice (C57BL/6 background; in-house) (39), aged 9–14 weeks and weighing 19–30 g, were used for all experiments (*n* = 4). Male and female mice were used in equal numbers. The mice were anesthetized with isoflurane and bled through the inferior vena cava on 0.109 M sodium citrate before being euthanized. Whole blood clots from WT and fibrinogen-deficient mice were prepared for SEM as described above, but clotting was initiated with mouse thrombin (0.5 U/ml). Each clot was formed in duplicate and imaged in 5 areas at 5 different magnifications (2,500×, 5,000×, 10,000×, 25,000×, and 50,000×) using a Hitachi SU8230 high-performance CFE SEM.

### In vivo mouse dermal punctures.

C57BL/6 (in-house) or BALB/cJRj mice (Janvier Labs), aged 9–14 weeks and weighing 19–30 g, were used for all experiments. Male and female mice were used in equal numbers. The mice were anesthetized with isoflurane, and the abdomen was shaved and waxed. Six puncture wounds were created using a 2-mm biopsy punch (World Precision Instruments Ltd.) and were filled with blood from a tail vein. The injury was either left uncovered or immediately covered with a thin layer of oil to remove the air-liquid interface. After 60 minutes the animals were euthanized by cervical dislocation, and the area around the wound together with the clot for each condition was surgically removed and fixed in 4% paraformaldehyde (histology or immunohistochemistry, *n* = 4) or 2% glutaraldehyde (SEM, *n* = 4). The fixed tissues were dehydrated and embedded in paraffin. Consecutive 5-μm sections were cut and mounted. Sections were then stained with MBS to observe the sectioned wound; collagen appears in blue, erythrocytes in yellow, and fibrin in pink. Slides were observed under an Olympus BX40 Dual View microscope, and photographs were taken using Image Pro-Plus 8.0 software. For immunohistochemistry, an EXPOSE rabbit specific HRP/DAB detection IHC kit (ab80437, Abcam) was used following the manufacturer’s instructions. Slides were stained with mouse anti-fibrin antibody (59D8, 1:1,000; antibody provided by Charles Esmon, Oklahoma Medical Research Foundation, Oklahoma City, Oklahoma, USA), which detects mouse fibrin (40), for 1 hour at room temperature in a humidity-controlled chamber. The sections were washed and incubated with HRP-conjugated anti-mouse secondary antibody. Negative controls were stained simultaneously in the absence of primary antibody. Alternatively, after fixing, samples were prepared for SEM as described above for clots.

### Red blood cell retention assay.

Red blood cell retention was measured by analyzing the quantity of hemoglobin released from different types of whole blood clots. Whole blood clots were formed in a well of an uncoated 8-well Ibidi slide, in the presence of CaCl_2_ (5 mM), with the addition of 1 pM tissue factor and incubated in a humidity chamber for 2 hours at room temperature. Clots were formed with no intervention, in the presence of Tween-20 (0.1%), or with the film being perforated after 1 hour. After 2 hours saline solution was added to the clot surface, and the clots were placed on an orbital shaker (400 rpm) for 30 minutes. A sample of the solution above the clot surface was taken and was diluted by 50% in distilled water and left for 30 minutes for hemolysis to occur. The samples were then analyzed for hemoglobin levels using the Harboe method and a spectrophotometer (41).

### Wound infection model.

Bioluminescent *P*. *aeruginosa* (strain Xen 41, derived from the parental pleural isolate PAO1; PerkinElmer), possessing a copy of the luxCDABE operon of *P*. *luminescens*, integrated at a single site on the chromosome, were aerobically grown in Todd Hewitt (TH) broth at 37°C to logarithmic phase (OD_620_, ~0.5). Bacteria were harvested, washed in PBS, and diluted in the same buffer to 2 × 10^8^ CFU/ml.

BALB/cJRj mice (8 weeks of age; Janvier Labs) were maintained under specific pathogen–free conditions and had free access to commercial chow and water. Male and female mice were used in equal numbers. For the experimental procedures, animals were anesthetized with isoflurane, and one puncture wound was created using a 2-mm biopsy punch (World Precision Instruments Ltd.) on the back of each mouse. The puncture wound was filled with blood from a BALB/cJRj donor mouse, and the wound was either left to clot while exposed to the air for 30 minutes (experimental group 1, *n* = 8) or immediately covered with mineral oil and left to clot for 30 minutes before the oil was washed off the clot with saline (experimental group 2, *n* = 8). As a control to investigate the effect of oil on bacterial proliferation, blood was not added to the puncture wounds on some mice, and these were either left untreated (control group 1, *n* = 4) or covered with mineral oil (control group 2, *n* = 4). All animals were subsequently infected with 2 μl bioluminescent *P*. *aeruginosa* suspension deposited on top of the clots. Mice were then anesthetized and imaged to check that the same amount of bacteria was added to each mouse and then anesthetized again at 4, 8, and 12 hours after bacterial infection to allow for data acquisition using a 3D IVIS Spectrum In Vivo Imaging System (PerkinElmer) and analysis using Living Image software (PerkinElmer). Differences in appearance between mice with film and without film in Figure 7 are due to the oil used to prevent film formation being transferred to the fur of the mice.

### Determination of bacterial CFU.

In order to study bacterial growth and dissemination, the skin around the wound was harvested from sacrificed animals at the end of the experiment (12 hours). The tissues were mechanically homogenized using 1.4-mm ceramic beads (QIAGEN) and a MagNA Lyser (Roche), and serial dilutions were subsequently plated on TH agar plates overnight at 37°C in order to enumerate the bacterial CFU present in the samples.

### Statistics.

All statistical analyzes were performed with GraphPad Prism 7. All experiments were repeated at least 3 times. Data were tested for normality using Shapiro-Wilk normality test. Data are presented as mean and SD for parametric data and mean and IQR for nonparametric data. For the comparisons of 2 groups, 2-tailed unpaired *t* test was performed. For comparisons between multiple groups, either 1-way ANOVA followed by Dunnett’s multiple comparisons test or 2-way ANOVA for parametric data was performed, and Kruskal-Wallis test was performed for nonparametric data. A *P* value less than 0.05 was considered significant.

### Study approval.

Ethical approval for blood taking was obtained from the University of Leeds Medical School or the University Hospitals of Geneva and Faculty of Medicine review board. Written informed consent was received from each patient and volunteer prior to inclusion in the study in accordance with the Declaration of Helsinki. Written informed consent was provided by the patient for the photograph shown in Supplemental Figure 9. All mouse experiments were conducted according to institutional guidelines and were authorized by either the University of Leeds Ethics Committee, in accordance with Home Office UK Animals (Scientific Procedures) Act 1986, or the Malmö-Lund Animal Care Ethics Committee, Sweden (entry no. M89-16).

## Author contributions

FLM and RASA conceived the project. FLM, RASA, CD, PP, SRB, KJK, JK, AC, SDC, HP, ASW, and HH contributed to design of the project, discussion, and interpretation of results. FLM performed most of the experiments unless stated otherwise. CD and HRM expressed mutant recombinant fibrinogens. CD, PP, and NY designed and performed murine experiments. SRB designed, performed, and analyzed AFM experiments. KJK performed Affimer experiments, SDS-PAGE, and Western blot experiments. SDC and NA helped design and perform Langmuir-Blodgett trough experiments. AC provided dysfibrinogenemia and afibrinogenemia patient samples. JLD provided fibrinogen-deficient mice. FLM, CD, and RASA wrote the manuscript. All authors critically reviewed the manuscript.

## Supplementary Material

Supplemental data

Supplemental Video 1

Supplemental Video 2

Supplemental Video 3

Supplemental Video 4

Supplemental Video 5

Supplemental Video 6

Supplemental Video 7

Supplemental Video 8

Supplemental Video 9

## Version 1 05/03/2018

In-Press Preview

## Version 2 06/25/2018

Electronic publication

## Version 3 08/01/2018

Print issue publication
