# Supplementary material for: A fibrin biofilm covers blood clots and protects from microbial invasion
Source: J Clin Invest. 2018 Jun 25;128(8):3356–68. doi: 10.1172/JCI98734 (PMC6063501; doi:10.1172/JCI98734)
Supplement: Supplemental data [file jci-128-98734-s012.pdf]

A fibrin biofilm covers the blood clot and protects from microbial invasion

Fraser L. Macrae[1†], Cédric Duval[1†], Praveen Papareddy[2], Stephen R. Baker[1], Nadira Yuldasheva[1], Katherine J. Kearney[3], Helen R. McPherson[1], Nathan Asquith[1], Joke Konings[4,5], Alessandro Casini[6], Jay L. Degen[7], Simon D. Connell[8], Helen Philippou[1], Alisa S. Wolberg[9], Heiko Herwald[2], Robert A. S. Ariëns[1,4,*]

Affiliations:

1. Thrombosis and Tissue Repair Group, Discovery and Translational Science Department, Leeds Institute of Cardiovascular and Metabolic Medicine, School of Medicine, University of Leeds, Leeds, UK.
2. Division of Infection Medicine, Department of Clinical Sciences, Faculty of Medicine, Lund University, Lund, Sweden;
3. Population and Clinical Sciences Department, Leeds Institute of Cardiovascular and Metabolic Medicine, School of Medicine, University of Leeds, Leeds, UK.
4. Department of Biochemistry, Cardiovascular Research Institute Maastricht, School of Medicine, University of Maastricht, Maastricht, the Netherlands
5. Synapse Research Institute, CARIM, University of Maastricht, Maastricht, the Netherlands.
6. Division of Angiology and Haemostasis, Faculty of Medicine, Geneva University Hospitals, Geneva, Switzerland.
7. Cincinnati Children's Hospital, Cincinnati, OH, USA
8. Molecular and Nanoscale Physics Group, School of Physics and Astronomy, University of Leeds, Leeds, UK.
9. Department of Pathology and Laboratory Medicine, University of North Carolina, Chapel Hill, NC, USA

† These authors contributed equally to this study.

The authors have declared that no conflict of interest exists.

This work is licensed under the Creative Commons Attribution 4.0 International License. To view a copy of this license, visit http://creativecommons.org/licenses/by/4.0/.

*Correspondence to:
R A S Ariëns, PhD
LIGHT Laboratories
University of Leeds
Clarendon Way
Leeds, LS2 9JT
UK
Email: r.a.s.ariens@leeds.ac.uk
Tel: +44 113 343 7734.

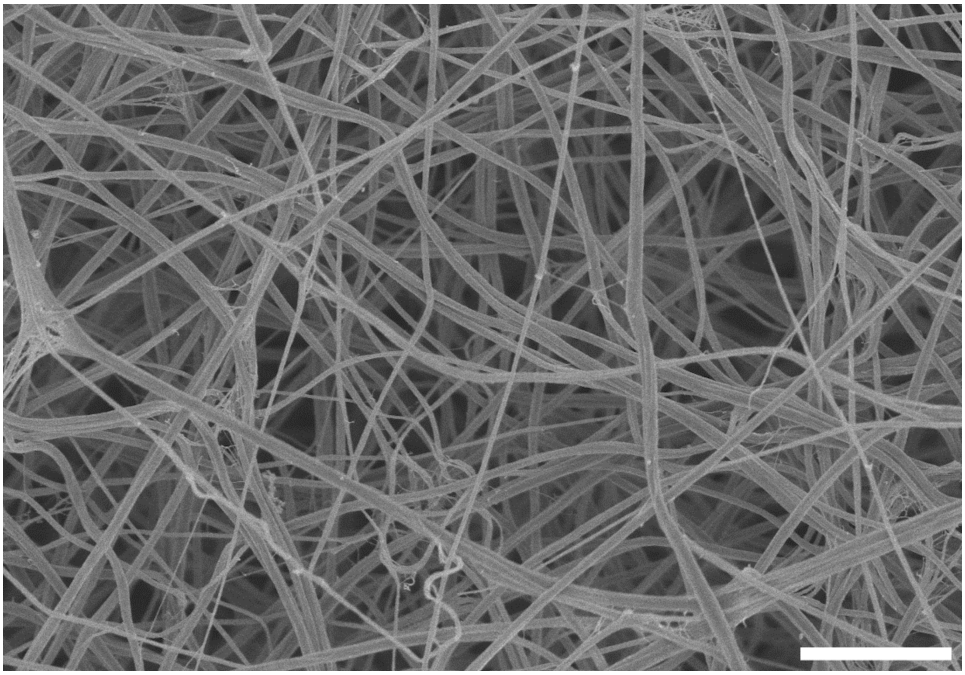

Supplemental Figure. 1. Fibrin clot network. SEM image of a three-dimensional fibrin fibre network from the inside of a clot underneath the air-liquid interface. Fibrin fibres in this image appear limitless and without fibre ends. Scale bar - 2μm

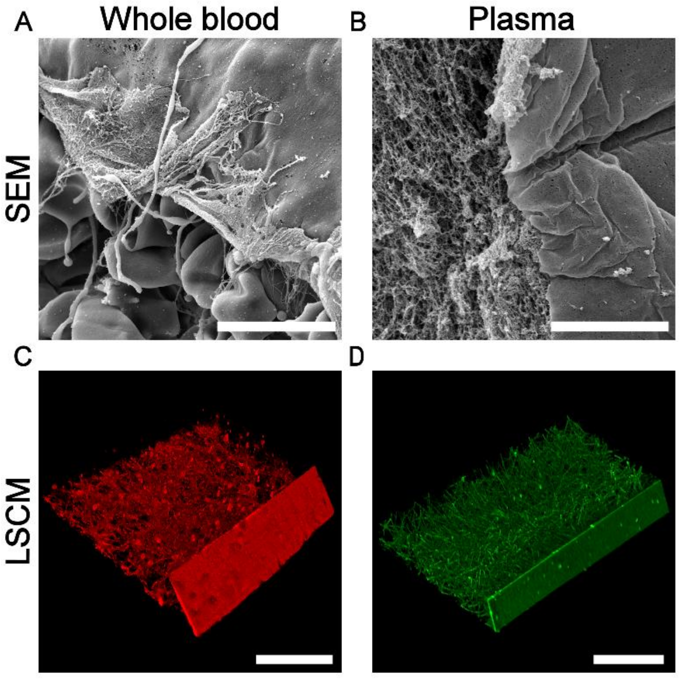

Supplemental Figure. 2. Film forms over surface of clots at air-liquid interface with tissue factor. **A, B**, SEM images of fibrin film formed at the air-liquid interface in whole blood and plasma in which clotting is triggered with tissue factor. Scale bar – A 20µm, B 50µm. Images representative of n=3 individuals **C, D**, LSCM of film formed at the air-liquid interface in whole blood and plasma clots triggered with tissue factor. Fibrinogen was fluorescently labelled with Alexa Fluor-488 (green) or -594 (red). Scale bar – C and D 50µm. Images representative of n=3 individuals.

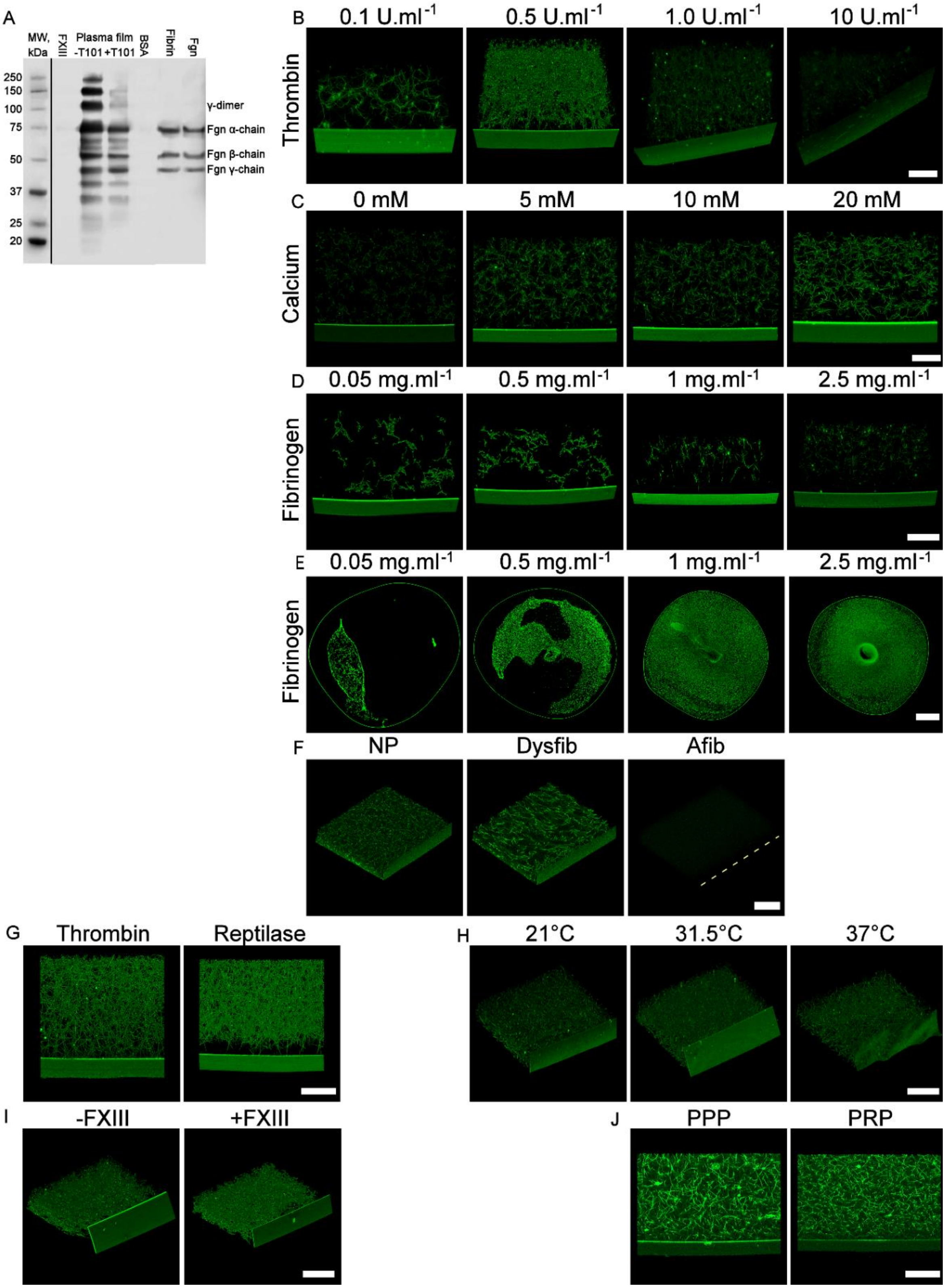

Supplemental Figure. 3. Fibrin film formation in different conditions. **A,** The film was removed from clots formed with or without T101, and run alongside FXIII, BSA, a reduced purified fibrin clot and purified fibrinogen on reducing SDS-PAGE and analysed by western blotting with a polyclonal antibody against fibrinogen. Representative blot of n=3 experiments. Fgn – purified fibrinogen, BSA – bovine serum albumin, MW – molecular weight marker. **B-J,** Representative images (n=4-6 experiments) of 3D z-stack images obtained with LSCM of clots produced with purified fibrinogen in different conditions. **B,** initiated with different thrombin concentrations, **C,** with different $CaCl_2$ concentrations (note that the image for 5mM is the same as figure 2D), **D,** with different fibrinogen concentrations, **E,** single z-plane slice of plasma clots with different fibrinogen concentrations showing a continuous film around the whole clot even when there is insufficient fibrinogen to fill the whole droplet (note that the image for 1 $mg.ml^{-1}$ is from the same sample as figure 1E), **F,** representative images of normal pool (NP, n=3 experiments) v dysfibrinogenemia (Dysfib, n=2 patients)/afibrinogenaemia (Afib,n=3 patients), **G**, initiated with thrombin or reptilase, **H**, clots formed at different temperatures, **I**, with or without FXIII, **J**, platelet poor plasma (PPP) v platelet rich plasma (PRP), n=6 individuals. Scale bars - 50µm.

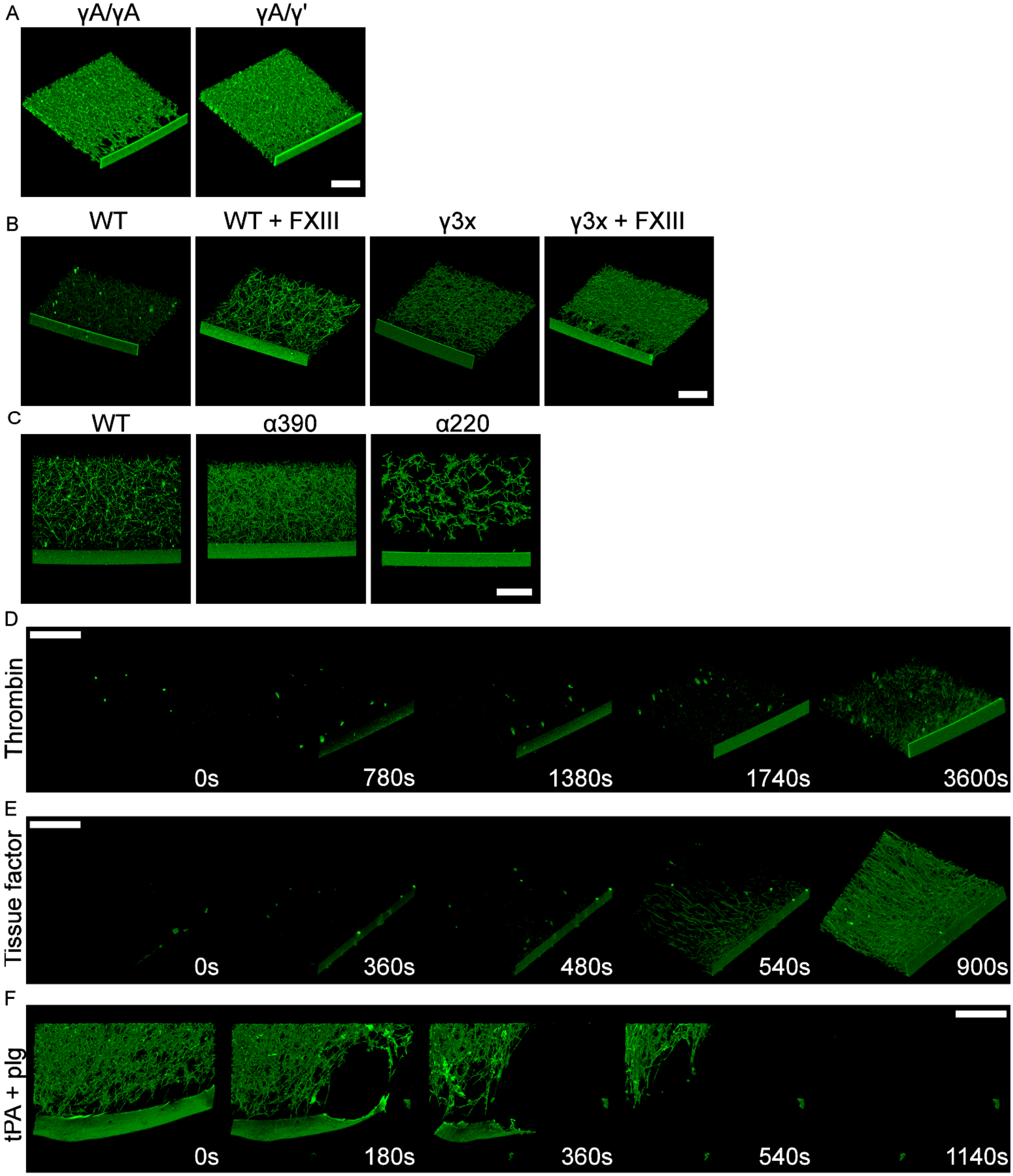

Supplemental Figure. 4. **Fibrin film formation and breakdown**. **A-C,** Representative images (n=4-6 experiments) of LSCM 3D z-stack images of clots produced with different purified fibrinogen variants. **A,** Formed with either γA/γA or γA/γ' fibrinogen. **B,** Clots were formed with recombinant wildtype fibrinogen or *γ*3x fibrinogen with or without FXIII **C,** Clots were formed with recombinant wildtype fibrinogen or mutant α220 or α390 fibrinogens. **D,** Clotting with purified fibrinogen was initiated with thrombin and followed over time. **E,** Clotting was initiated with tissue factor in plasma and followed over time. **F,** Fully formed clots produced with purified fibrinogen were broken down with tPA and plasminogen and followed over time. D-E n=3 experiments. Scale bars – 50μm. WT – wild type, plg – plasminogen.

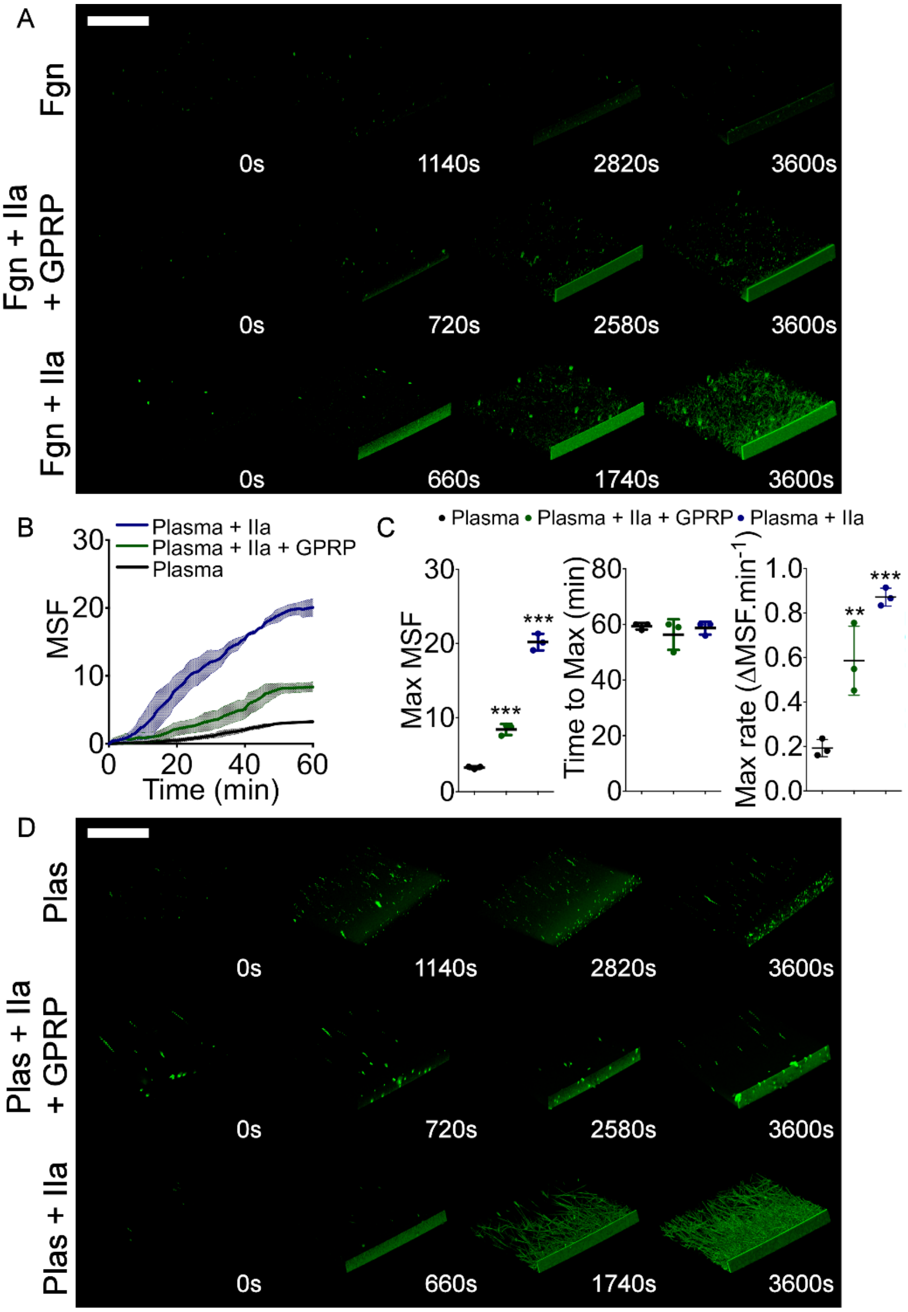

Supplemental Figure. 5. Fibrin(ogen) accumulation at the air liquid interface by LSCM. **A,** Accumulation of fibrinogen, fibrin monomers (fibrinogen + IIa + GPRP) or polymerizing fibrinogen at the air-liquid interface in a purified system over time. Images representative of n=3 experiments **B,** Accumulation of MSF of plasma clots over time as analysed by LSCM, data shown as mean ± SD. (n=3 experiments) **C,** Max MSF, F=370.2, df=2, P=< 0.0001, time to max MSF, F=0.6036, df=2, P=0.5770 and maximum rate of MSF increase, F=38.51, df=2, P=0.0004, data shown as mean ± SD, n=3 experiments. * represents difference from black bar, ** P<0.01, *** P<0.001. **D,** Accumulation of fibrinogen, fibrin monomers (fibrinogen + IIa + GPRP) or polymerizing fibrinogen at the air-liquid interface in a plasma system over time. Images representative of n=3 experiments (note that images for Plas + IIa are from the same sample as figure 3A). MSF – mean sheet fluorescence, IIa – thrombin, GPRP - Gly-Pro-Arg-Pro peptide, Fgn – fibrinogen, Plas – Plasma. Scale bars - 50µm.

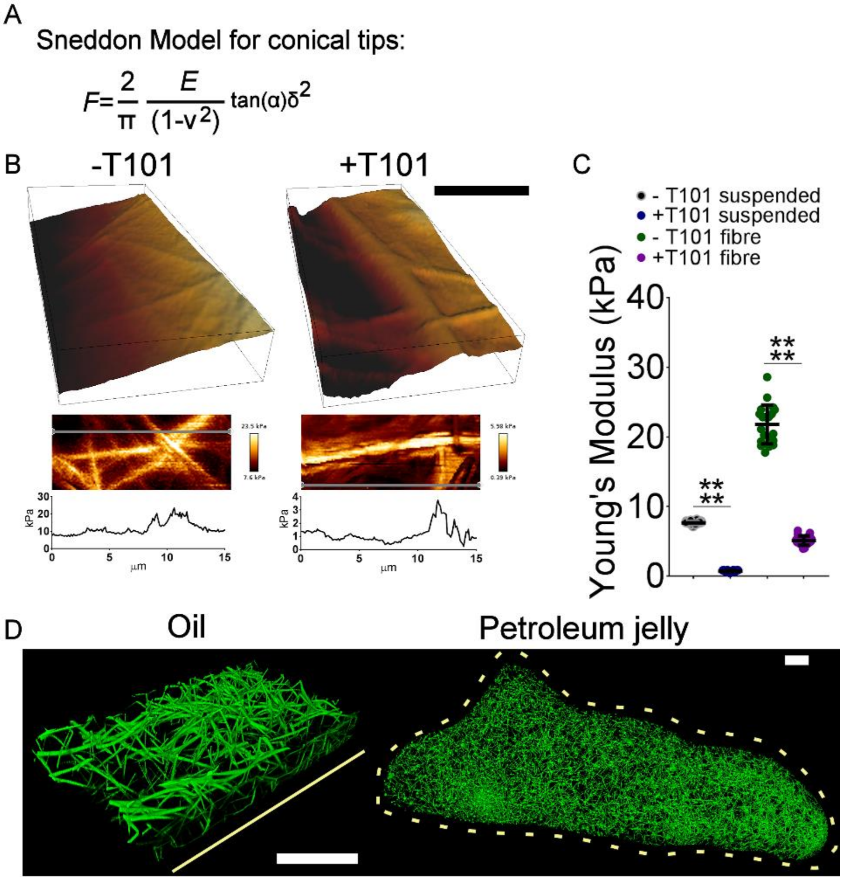

Supplemental Figure. 6. Mechanisms and roles of fibrin film. **A,** Sneddon model used to calculate Young's Modulus, where F is the force from the force curve, E is Young's modulus, v is Poisson's ratio (0.5), α is the half angle for the indenter (15 degrees for our tips), and δ is the indentation. Note that this equation is only accurate with a half angle of 15 degrees for the first 200nm of indentation. **B,** Strength of the fibrin film in clots produced with plasma and thrombin with or without T101 (FXIII inhibitor) investigated using atomic force microscopy (AFM). Fibrin fibres were visible under the film surface and these areas presented with stiffer Young's modulus than fibrin film suspended between fibres. Grey lines in the zoomed-in images represent Young's modulus scan area represented in the line force graphs. Scale bar - 2µm. **C,** Young's Modulus was calculated for the suspended film and the film supported by fibers with and without T101 by fitting a Sneddon model to all AFM force curves found over the entire area that was imaged. 20 measurements were taken for each condition. **** P<0.0001. **D,** Clots produced from plasma with thrombin, under a layer of oil or enclosed in a ball of petroleum jelly, to eliminate the air-liquid interface, imaged by LSCM. Solid and dotted yellow lines indicate location of air liquid interface, n=3 experiments. Scale bars - 50µm.

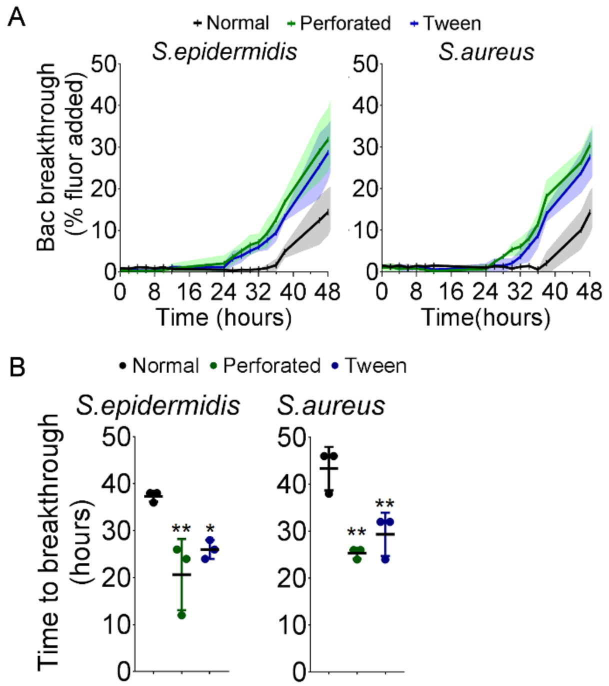

Supplemental Figure. 7. Effects of fibrin film on bacteria migration. A, Movement of fluorescently labelled *S. aureus* and *S. epidermidis* bacteria through the clots (displayed as quantity of the fluorescent bacteria breaking through the clot, as a percentage of fluorescent bacteria added) with three different film conditions; normal, perforated or removal with tween-20. Bac – bacteria. **B,** Time taken for the first fluorescently labelled *S. aureus* and *S. epidermidis* bacteria to break through the clot. * represents difference from normal clot. * $P<0.05$, ** $P<0.01$, n=3 experiments, one-way ANOVA, *S.aureus* - F=18.27 , df=2, P=0.0028; *S.epidermidis* - F=10.4 , df=2, P=0.0112.

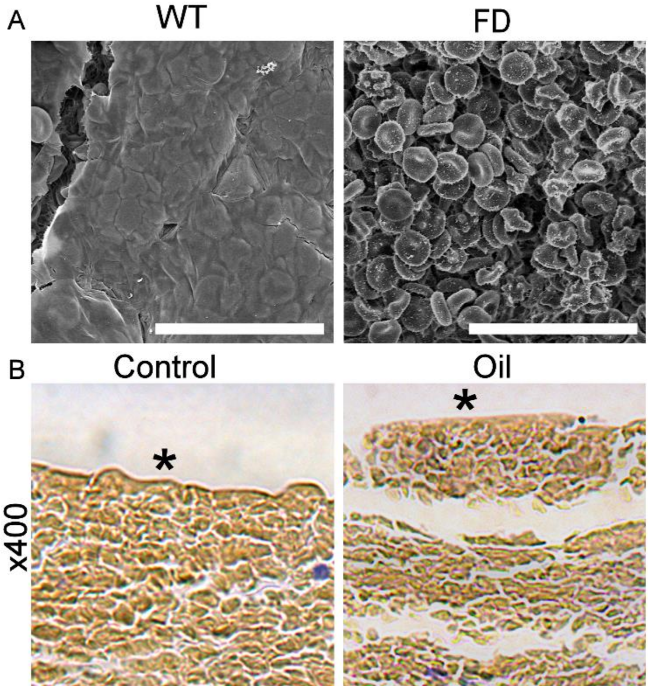

Supplemental Figure. 8.Murine dermal injury model. **A,** Clots formed using whole blood and murine thrombin from wildtype and fibrinogen-deficient mice were imaged by SEM. Images representative of n=4 mice. Scale bars- 20µm. FD – fibrinogen deficient. **B,** Clots from the murine dermal puncture model with and without oil, stained with mouse anti-fibrin antibody (59D8). In the absence of oil a brown layer can be seen along the surface of the clot, showing fibrin is present in the film. This layer was absent in clots formed with oil. * highlights air liquid interface. Images representative of n=4 mice.

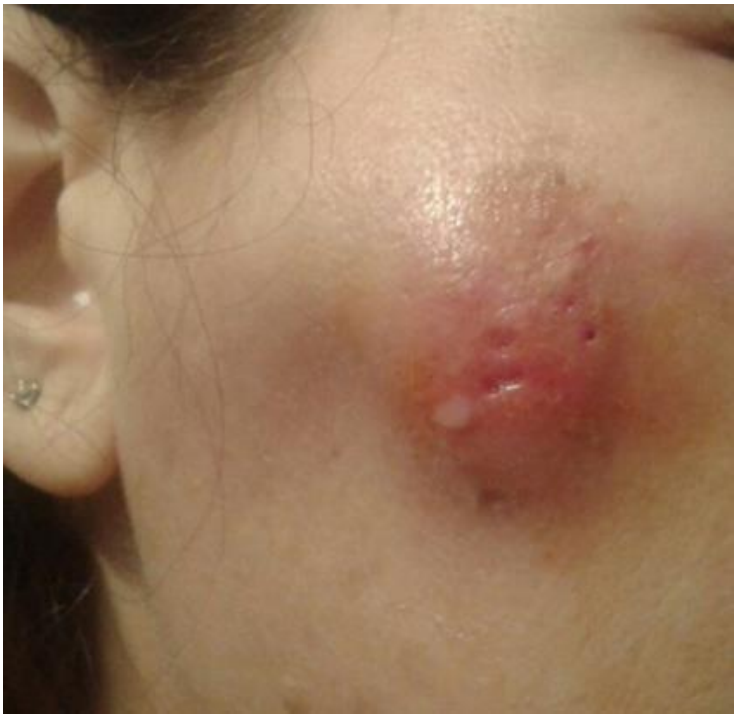

Supplemental Figure. 9. Dermal infection in a patient with afibrinogenemia.

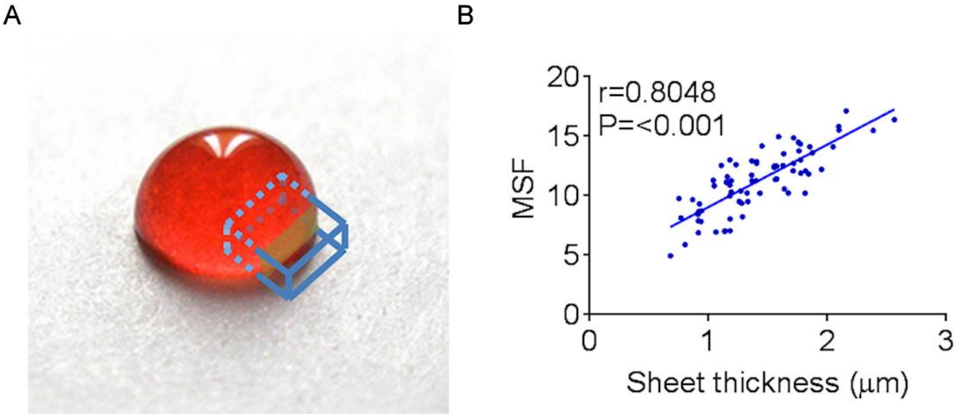

Supplemental Figure. 10. Laser Scanning Confocal Microscopy imaging. **A,** Diagrammatic representation of areas imaged during LSCM imaging **B,** Mean sheet fluorescence was validated by plotting MSF against sheet thickness, Pearson's correlation, n=72 experiments. MSF – Mean Sheet Fluorescence.

Supplemental Table 1. Mean sheet fluorescence and film thickness of fibrin films in different conditions

	Mean Sheet fluorescence					Estimated film thickness				
Condition	Mean	SD	p value	F/t	df	Mean (µm)	SD	p value	F/t	df
Thrombin ($U.ml^{-1}$)										
0.1	15.28	0.89	< 0.0001	193.3	3	2.15	0.40	0.0013	14.51	3
0.5	10.54	0.97				1.72	0.11			
1.0	7.57	0.68				1.16	0.11			
10	2.57	0.38				1.18	0.03			
Calcium (mM)										
0	6.24	1.02	0.003	8.58	3	0.87	0.24	0.026	5.34	3
5	12.69	3.45				1.54	0.28			
10	14.68	3.10				1.92	0.19			
20	11.83	1.33				1.3	0.51			
Fibrinogen ($mg.ml^{-1}$)										
0.05	9.34	0.83	<0.0001	23.69	3	1.85	0.17	<0.0001	15.81	3
0.5	7.59	1.03				1.70	0.19			
1.0	4.72	1.30				1.25	0.16			
2.5	5.05	1.19				1.41	0.14			
Normal pool	10.00	1.57				1.75	0.11			
Dysfibrinogenemia	13.24	0.71				1.99	0.14			
Afibrinogenemia	0.22	0.24				0.00	0.00			
Thrombin (0.5 U.ml-1)	12.3	0.94	0.057	2.36	6	1.75	0.17	0.8	0.27	6
Reptilase (2.4 U.ml-1)	14.06	1.16				1.84	0.51			
21°C	10.23	0.73	< 0.0001	34.96	2	1.19	0.28	0.016	9.03	2
31.5°C	12.67	0.75				1.54	0.15			
37°C	8.54	0.63				0.88	0.10			
-FXIII	13.14	1.25	0.82	0.24	6	1.56	0.33	0.65	0.5	6
+FXIII (3.7 µg.ml-1)	12.69	3.45				1.71	0.41			
PPP	13.51	2.35	0.26	1.19	10	1.58	0.29	0.27	1.18	10
PRP	12.06	1.81				1.42	0.16			
$\gamma A/\gamma A$	10.61	1.29	0.49	0.72	6	1.17	0.11	0.54	0.68	6
$\gamma A/\gamma'$	11.23	1.66				1.12	0.07			
WT	9.21	1.83	0.007	6.49	3	0.92	0.01	0.0002	25.58	3
WT + FXIII	9.08	2.44				1.1	0.16			
γ3x	12.69	1.04				1.51	0.09			
γ3x + FXIII	13.05	1.04				1.51	0.09			
WT	9.08	1.25	< 0.0001	28.78	2	1.1	0.16	0.037	6.03	2
α220	16.24	1.99				1.73	0.37			
α390	11.74	1.63				1.21	0.09			

df – degrees of freedom, F/t – F or t value for ANOVA or t-test, FXIII – factor XIII, MSF – mean sheet fluorescence, SD – standard deviation, WT – wildtype

Supplemental Table 2. Effects of fibrinogen and fibrin quantity on time to first increase in surface pressure

No. of molecules ($x10^{13}$)	Fibrinogen		Fibrin	
	Time (s)	SD	Time (s)	SD
1	N/A		N/A	
5	918.0	140.3	1018.0	31.0
20	519.3	22.2	647.0	60.2
30	32.3	19.9	34.0	18.0
100	24.0	5.2	24.0	5.0
5312	9.0	0.0		

N/A – not applicable (surface pressure did not increase), SD – standard deviation

Supplemental Table 3. Maximum surface pressure

	Fibrinogen		Fibrin				
No. of molecules (x10^{13})	Max surface pressure (mN.m^{-1})	SD	Max surface pressure (mN.m^{-1})	SD	p value	t-value	df
1	0.48	0.05	0.43	0.02	>0.99	0.14	20
5	12.84	0.25	1.15	0.65	<0.0001	35.07	20
20	15.83	0.71	14.34	0.24	0.001	4.47	20
30	16.30	0.04	16.74	0.32	0.68	1.32	20
100	16.39	0.23	16.41	0.69	>0.99	0.06	20
5312	16.28	0.29					

Two way ANOVA, df – degrees of freedom, SD – standard deviation

Supplemental Table 4. Mean sheet fluorescence of fibrin(ogen) accumulation at air-liquid interface

Condition	Max MSF	SD	Time to Max MSF (s)	SD	Max rate of MSF increase (δMSF.min^{-1})	SD
Fibrinogen	5.5	1.3	3540	60	0.33	0.03
Fibrinogen + IIa + GPRP	10.4	0.8	3480	208	0.61	0.11
Fibrinogen + IIa	27.1	1.1	3540	104	1.46	0.39
Plasma	3.3	0.2	3560	69	0.19	0.04
Plasma + IIa + GPRP	8.4	0.8	3380	330	0.59	0.16
Plasma + IIa	20.2	1.1	3520	139	0.87	0.04

GPRP – Gly-Pro-Arg-Pro peptide, IIa – thrombin, MSF – mean sheet fluorescence, SD – standard deviation

Supplemental Table 5. Bacterial proliferation measured by bioluminescence

Condition	Bioluminescence x10^5 $p^{-1}.sec^{-1}.cm^{-2}.sr^{-1}$	IQR (25,75%)	p value	Kruskal-Wallis statistic	Number of groups
4 hours					
+ film	0.152	0.147, 0.236	>0.999	28.11	6
- film	0.285	0.196, 0.541			
8 hours					
+ film	0.193	0.165, 0.259	0.046		
- film	0.690	0.500, 2.605			
12 hours					
+ film	0.151	0.107, 0.371	0.002		
- film	1.034	0.470, 4.200			

IQR – Interquartile range

Movie legends:

Supplemental Movie 1. Film formation in plasma with thrombin
Supplemental Movie 2. Film formation with purified fibrinogen and thrombin
Supplemental Movie 3. Film formation in plasma with tissue factor
Supplemental Movie 4. Film lysis in plasma with tPA
Supplemental Movie 5. Lysis of a film formed from purified thrombin with tPA and plasminogen
Supplemental Movie 6. Accumulation of purified fibrinogen at the clot surface
Supplemental Movie 7. Accumulation of fibrin monomers at the clot surface
Supplemental Movie 8. Accumulation of fibrinogen in plasma at the clot surface
Supplemental Movie 9. Accumulation of fibrin monomers in plasma at the clot surface
